# Immunomodulatory peptides: new therapeutic horizons for emerging and re-emerging infectious diseases

**DOI:** 10.3389/fmicb.2024.1505571

**Published:** 2024-12-20

**Authors:** Debolina Chatterjee, Karthikeyan Sivashanmugam

**Affiliations:** School of Biosciences and Technology, Vellore Institute of Technology SBST, Vellore, Tamil Nadu, India

**Keywords:** host defense peptides, eukaryotes, HDPs, anti-inflammatory activity, immune response, MDR

## Abstract

The emergence and re-emergence of multi-drug-resistant (MDR) infectious diseases have once again posed a significant global health challenge, largely attributed to the development of bacterial resistance to conventional anti-microbial treatments. To mitigate the risk of drug resistance globally, both antibiotics and immunotherapy are essential. Antimicrobial peptides (AMPs), also referred to as host defense peptides (HDPs), present a promising therapeutic alternative for treating drug-resistant infections due to their various mechanisms of action, which encompass antimicrobial and immunomodulatory effects. Many eukaryotic organisms produce HDPs as a defense mechanism, for example Purothionin from *Triticum aestivum* plant, Defensins, Cathelicidins, and Histatins from humans and many such peptides are currently the focus of research because of their antibacterial, antiviral and anti-fungicidal properties. This article offers a comprehensive review of the immunomodulatory activities of HDPs derived from eukaryotic organisms including humans, plants, birds, amphibians, reptiles, and marine species along with their mechanisms of action and therapeutic benefits.

## 1 Introduction

Microbial evolution has given us many economically important microorganisms as well as pathogens. Evolution of antimicrobial resistance genes among the microbial strains has been taking place alarmingly over the past few years, leading to the emergence of multi-drug resistant (MDR) or extensively virulent and drug-resistant species such as the *Enterococcus faecium, Staphylococcus aureus, Klebsiella pneumoniae, Acinetobacter baumannii, Pseudomonas aeruginosa*, and *Enterobacter* spp., commonly termed the ESKAPE pathogens. This resulted in the discovery of next-generation alternative therapeutics known as host defense or antimicrobial peptides (AMPs) (Guryanova and Ovchinnikova, 2022). These peptides can be found in a wide variety of prokaryotic and eukaryotic organisms in nature. They are of short-length (~ 10 to 50 amino acids) peptides, mostly cationic with basic and hydrophobic amino acids (Huan et al., 2020). Many previous studies revealed that most of these cationic peptides were found to have microbicidal, cytotoxic and immunomodulatory activities against both harmful emerging and remerging pathogens like bacteria, protozoans, yeast, fungi and viruses (Luong et al., 2020; Pasupuleti et al., 2012).

**Figure 1:**
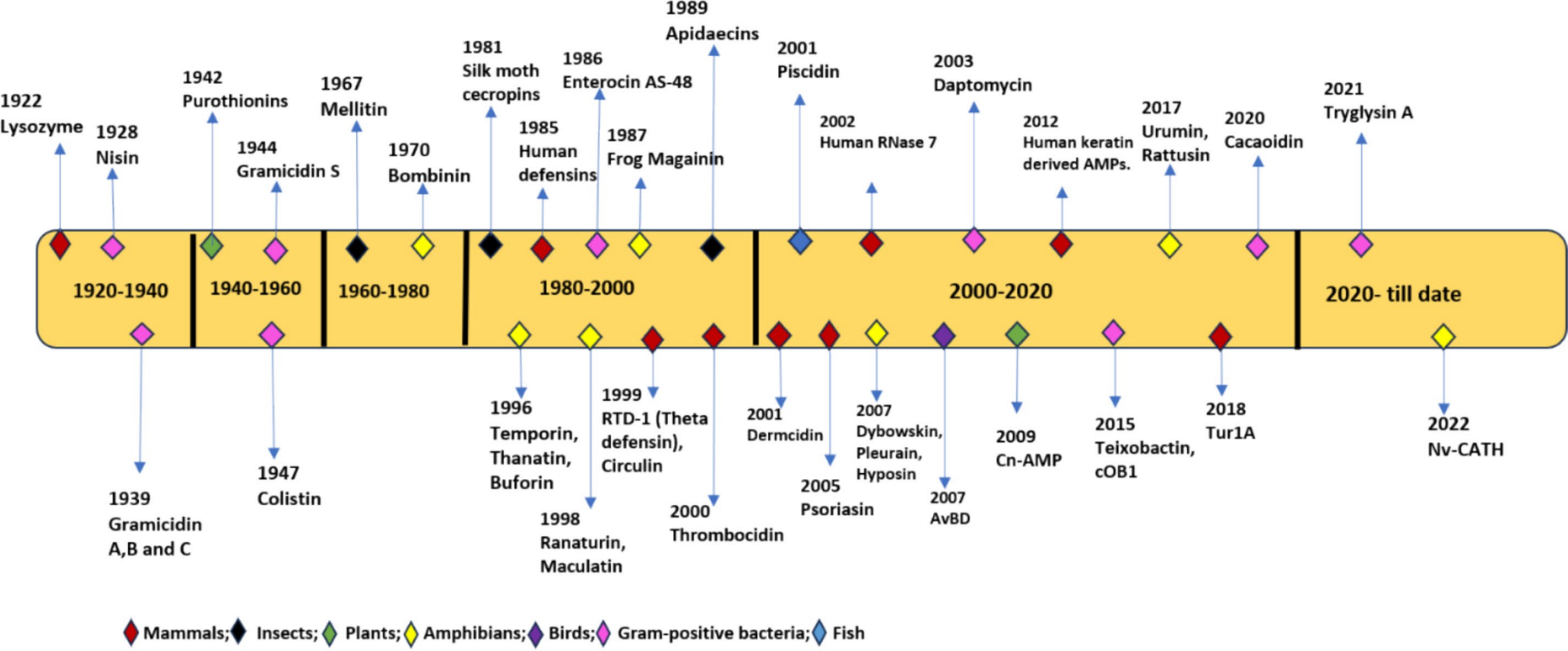
Chronological order of discovery of antimicrobial peptides (AMPs) from 1922 to 2022.

In 1939, Gramicidin was the first AMP isolated from *Bacillus* species having bactericidal activity against *S. pneumonia* in mice. This led to the discovery of many AMPs in both prokaryotes and eukaryotes including bactericidal tyrocidine from *Bacillus brevis*, Purothionin with fungicidal and bactericidal properties from *Triticum aestivum* plant (Fernandez de Caleya et al., 1972). In 1956, first animal AMP defensin was isolated from leukocyte cells of rabbits, followed by lactoferrin from cow’s milk (Kühnle et al., 2019), cecropins from hemolymph of butterfly pupae *Hyalophora cecropia* (Wu et al., 2003) and in 1986 Magainins from mucous membrane of frog *Xenopus laevis* (Zasloff, 1987). AMPs were also found in lysosomes of human leukocytes and the human female reproductive tract (Sharma et al., 2011). To accommodate the increasing number of AMPs, an antimicrobial peptides database was built in 2011. To date, more than 3,200 peptides from various sources, including amphibians (28%), birds (22%), arthropods (11%), plants (10%), insects (7.9%), bacteria (7.4%), mammals (humans) (3.0%), Pisces (2.5%), viruses (1.2%), and fungus (0.4%), have been deposited in the database (Jhong et al., 2022; Guryanova and Ovchinnikova, 2022). CAMPR3, is another database used in the identification of natural AMPs based on structural and sequence analysis, which can be used in designing new and efficient AMPs (Waghu et al., 2016). Figure 1 depicts the chronological order of discovery starting from 1929 till date as antimicrobial drugs.

The immunomodulatory action of small peptides to protect the hosts from infections has been extensively investigated in recent years. These can stimulate or inhibit the host immune system by targeting immune cells such as leukocytes, macrophages, neutrophils and mast cells, thus leading to wound healing and angiogenesis (Lesiuk et al., 2022). Defensins and cathelicidins with immunomodulatory functions have been identified in a variety of sources, including both porcine and human samples (Dlozi et al., 2022). Most of these immunomodulatory peptides were found to be cost-effective, safe and their therapeutic applications are still under process of discovery. The present article provides an overview of natural and synthetic-derived peptides with immunomodulatory activity from various sources to understand their structural and therapeutic properties.

## 2 Immunomodulatory mechanism of action

**Figure 2:**
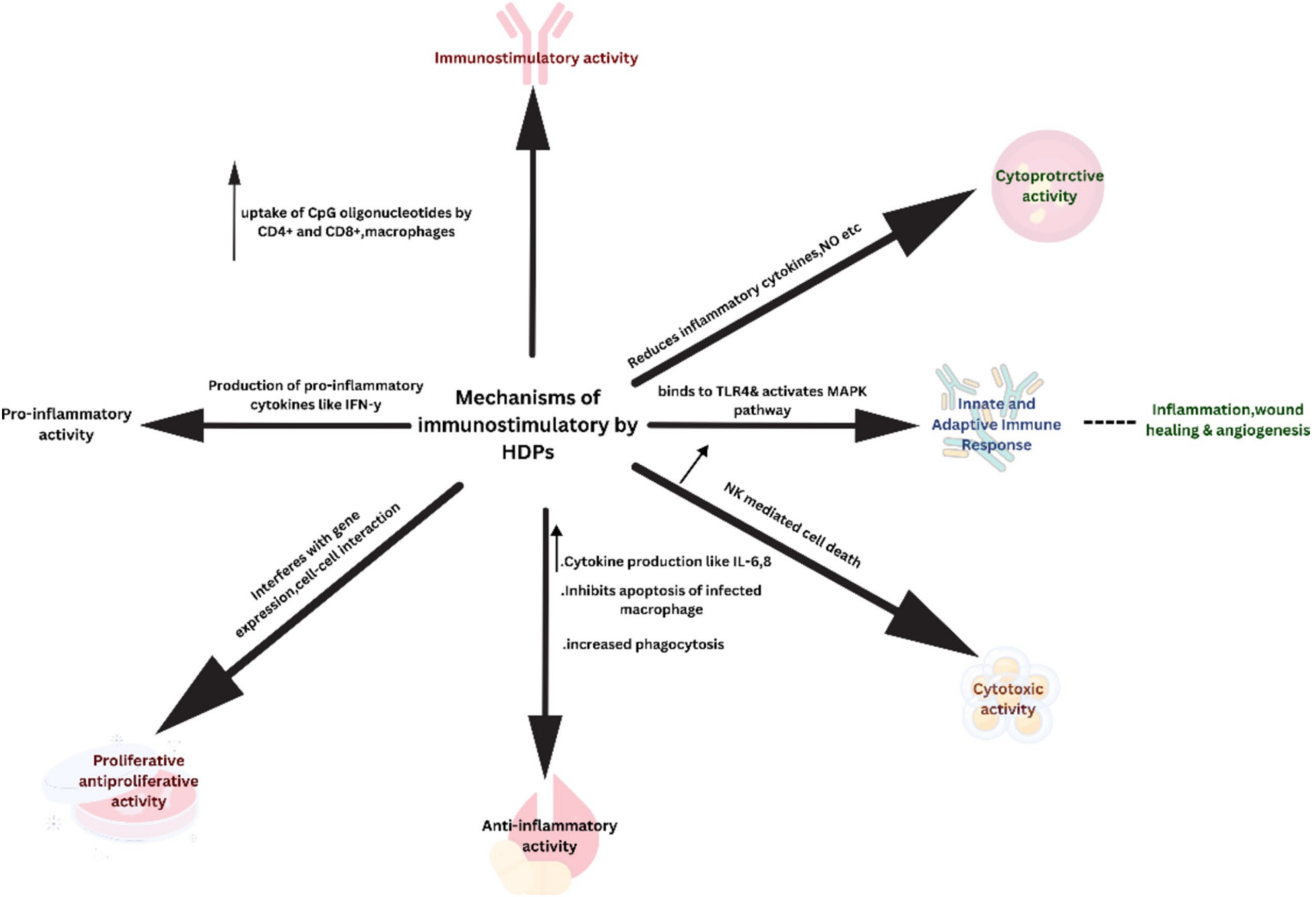
Immunomodulatory mechanisms of the host defense peptides.

The mechanism of immunomodulatory peptides mainly involves intracellular uptake of these peptides via membrane-bound G-protein receptors or localized translocation. They modulate signaling pathways by interacting intracellularly with signaling molecules or receptors (p62 and GAPDH) specifically targeting protein kinases to promote dendritic cell differentiation, recruitment of macrophages and mast cells inducing phagocytosis, stimulating secretion of anti-inflammatory cytokines, causing wound healing, apoptosis and lipopolysaccharide induced suppression of pro-inflammatory cytokines illustrated in Figure 2 (van der Does et al., 2019; Mookherjee et al., 2020; Barlow et al., 2010).

### 2.1 Recruitment of leukocytes

One of the primary immunomodulatory functions of HDPs was the stimulation of chemokine secretion. Also, they function as chemokines at high concentrations, thereby enhancing chemotactic activity and leukocyte recruitment (Nijnik et al., 2010; Rivas-Santiago et al., 2013). The underlying mechanisms involve multiple cellular chemokine receptors, including G-coupled protein, CCR6, CCR2, and Toll-like receptors, as well as contact with intracellular signaling proteins like GAPDH and p62, which allows eradication of the infections thereby promoting faster wound healing (Hancock et al., 2016; Choi and Mookherjee, 2012).

### 2.2 Modulation of inflammatory response

HDPs can modulate the pro-inflammatory response by suppressing cytokines production, including interleukins such as IL-6, IL-8 and TNF-*α*, IL-6, and IL-8 in response to lipopolysaccharides (LPS). The LL-37 peptide was found to modulate cytokine TNF-α production produced in response to lipoteichoic acid and lipo-polysaccharides. They have effectively inhibited pro-inflammatory genes (Overhage et al., 2008; von Köckritz-Blickwede et al., 2008). Similarly, these peptides function as anti-inflammatory agents by preventing the binding of inflammatory stimulators to their target receptors or molecules. This is achieved either by neutralizing lipopolysaccharides (LPS) or by means of competitive inhibition of LPS and CD14 binding. Apart from these, they can suppress the release of interleukins or the expression of transcription factors (Luo and Song, 2021; Rajasekaran et al., 2019).

### 2.3 Neutrophil function modulation

HDPs can modulate neutrophil function either directly through chemotactic activity or indirectly by triggering the release of chemokines such as Gro-*α* and IL-8 to control infections (Hemshekhar et al., 2018; Zheng et al., 2007). In addition, neutrophil-derived extracellular traps (NET) containing DNA and HDPs stored in primary and secondary granules of neutrophils destroy biofilms and bacterial growth (de la Fuente-Núñez et al., 2014).

### 2.4 Enhancement of adaptive immunity

HDPs are capable of recruiting the antigen-presenting cells (APCs) to the infection site, thereby establishing a link between innate and adaptive immunity (Yu et al., 2007; Davidson et al., 2004). In addition to activating APCs, cationic HDPs (CHDPs) possess the ability to regulate the lymphocyte responses, which in turn impacts the adaptive immune response (Allaker, 2008). By boosting immunological activity, these peptides have the potential to cause phagocytic removal of microorganisms (Conlon, 2015).

## 3 Naturally occurring host defence peptides

The following section summarizes the HDPs from various eukaryotic sources, such as humans, avians, reptiles, amphibians and marine organisms. A detailed list of the peptides from various sources along with their mechanism of action was given in Table 1.

**Table 1:** List of host defence peptides from various sources along with their structural details and mechanism of action.

Name of the peptide		Sequence	Source	Number of amino acids/Mol.wt	Secondary Structure composition analysis using SOPMA	PDB	Mechanism of action	References
Humans								
β-defensins	HBD1	DHYNCVSSGGQCLYSACPIFTKIQGTCYRGKACCK	_	35/3806.4323	Alpha Helix (45.71%) Extended Strand (34.29%) Beta turn (20.00%)	1KJ5	Chemoattractant for immature dendritic cells and memory T cells and mediates activity by a chemokine receptor CCR6.	Valore et al. (1998), Hoover et al. (2001), and Semple and Dorin (2012)
	HBD2	TCLKSGAICHPVFCPRRYKQIGTCGLPGTKCCKKP	_	35/3795.6725	Extended Strand: 2.86% Beta turn: 5.71% Random coil: 91.43%	1FD3	Pro-inflammatory; expressed in atopic dermatitis, GI infection at both mRNA and protein level.	Schröder and Harder (1999) and Koeninger et al. (2020)
α-defensins	HNP1	ACYCRIPACIAGERRYGTCIYQGRLWAFCC	_	30/3448.1296	Alpha helix (3.33%); Extended strand (43.33%); Beta turn (20.00%) and Random coil (33.33%) 1 is 3.33%	3GO0	Pro-inflammatory and inhibits differentiation of monocytes; potent chemoattractant that HNP2	Brook et al. (2016), Wang et al. (2016), and Bowdish et al. (2006)
	HNP2	CYCRIPACIAGERRYGTCIYQGRLWAFCC	_	29/3377.0508	Extended strand: 44.83% Beta turn: 13.79% Random coil: 41.83%	1ZMH	Induces chemokine activity in monocytes	Lehrer and Lu (2012), Wang (2014), and Pachón-Ibáñez et al. (2017)
	HNP4	CYCRIPACIAGERRYGTCIYQGRLWAFCC	_	29/3377.0508	Extended strand: 44.83% Beta turn: 13.79% Random coil: 41.83%	6DMM	found in neutrophils and have corticostatic activity.	Xu and Lu (2020) and Bowdish et al. (2006)
	HDP5	ATCYCRHGRCATRESLSGVCEISGRLYRLCCR	_	32/3624.2628	Alpha helix: 46.88% Extended strand: 15.62% Beta turn: 12.5% Random coil: 25%		Expressed at high concentration in jejunum and ileum, level increases during acute coeliac sprue and decreases in HIV	Petkovic et al. (2021) and Hancock et al. (2016)
	HDP6	ATCYCRHGRCATRESLSGVCEISGRLYRLCCR	_	32/3624.2628	Alpha helix: 46.88% Extended strand: 15.62% Beta turn: 12.5% Random coil: 25%		Expressed at high concentration in jejunum and illeum and level increases during acute coeliac sprue and decreases in HIV.	
Cathelicidin/LL-37		LLGDFFRKSKEKIGKEFKRIVQRIKDFLRNLVPRTES	_	37/4493.3271	Alpha helix (91.89%); Random Coil: 5.41% Beta turn: 2.7%	2FBS	Pro/Anti-inflammatory and allows chemotactic regulation of monocytes	Sandra Tjabringa et al. (2005), Nell et al. (2006), and Chen et al. (2013)
Pep19-2.5		GCKKYRRFRWKFKGKFWFWG	_	20/2712.2721	Extended strand: 65.00%;Beta turn: 15.00%; Random coil: 20%		Mostly anti-inflammatory	Heinbockel et al. (2021)
IDR-1018		VRLIVAVRIWRR	_	12/1536.9339	Random Coil (100%)		Pro/anti-infalmmatory and causes macrophage activation	Mansour et al. (2015)
Plants								
Cyclotide Cliotide	T28	GGSIPCGESCVFLPCFLPGCSCKSSVCYLN	*Clitoria ternatea* L.	30/3071.65	Extended strand: 40% Random coil: 50% Beta turn: 10%		*Invitro* increase in anti-inflammatory interleukines IL-8, IL6, TNF-α in macrophages	Gilding et al. (2016)
	T32	GDLFKCGETCFGGTCYTPGCSCDYPICKNN		30/3197.6440	Extended strand: 6.67% Beta turn: 13.33% Random coil:80%			Serra et al. (2016)
	T33	GFNSCSEACVYLPCFSKGCSCFKRQCYKN		29/3273.8345	Extended strand: 24.14% Beta turn: 17.24% Random coil: 58.62%			Nguyen et al. (2011)
Zein hydrolysates	Peptide 1	PFNQL	*Zea mays* L.	5/617.7026	Random coil: 100%		*Invitro* analysis showed inhibition of interleukin IL—6 in human cell line U937	Liu et al. (2020)
	Peptide 2	FLPFNQL		7/878.0387	Random coil: 100%			
	Peptide 3	SQLALTNPT		9/944.0527	Random coil: 100%			
	Peptide 4	GAPFNQ		6/632.6740	Random coil: 100%			
	Peptide 5	FLPPVT		6/672.8223	Random coil: 100%			
LR13		LLPPFHQASSLL	*Oryza sativa* L.	12/1322.5704	Random coil: 100%		*Invitro* and *invivo* anti-inflammatory response. *Invitro* downregulated IL-1β expression in macrophages, *invivo* upregulated IL-4 and IL-10 in CD4+ and CD25+ cells	Shapira et al. (2010)
α-Gliadin (pepsin-trypsin digested fragment)		PPYCTIVPFGIFGTNYR	Glutein containing grains	17/1945.2725	Extended strand: 29.41% Random coil: 70.59%		Bind to chemokine receptor CXCR3 and induced the release of IL-8 in coeliac patients	Lammers et al. (2011)
Amphibians- Frogs								
Frenatin-2D		DLLGTLGNLPLPFI	*Discoglossus sardus* (Tyrrhenian painted frog)	14/1482.7838	Random coil: 100%		Mouse peritoneal macrophages were stimulated by the peptide to release proinflammatory cytokines TNF- and IL-1. Also, both LPS-stimulated and unstimulated cells were stimulated for the production of IL-12.	Conlon et al. (2013)
Plasticin-L1		GLVNGLLSSVLGGGQGGGGLLGGIL	*Leptodactylus laticeps* (South-American Santa Fe frog)	25			Peritoneal macrophages from C57BL/6 and BALB/c mice produced increased proinflammatory interleukins IL-1β, IL-12, IL-23, and TNF-α.	Scorciapino et al. (2013)
Pseudhymenochirin	1Pb	IKIPSFFRNILKKVGKEAVSLIAGALKQS	*Pseudhymenochirus merlini*	29/3156.8505	Alpha helix: 75.86% Beta turn: 13.79% Random coil: 10.34%		Upregulation of proinflammatory IL-23 production and downregulation of anti-inflammatory IL-6 and IL-10 production by LPS-stimulated macrophages from mice	Mechkarska et al. (2014)
	2 Pa	GIFPIFAKLLGKVIKVASSLISKGRTE		27/2873.5199	Alpha helix: 70.37% Beta turn: 11.11% Random coil: 18.52%			
Magainin-AM1		GIKEFAHSLGKFGKAFVGGILNQ	*Xenopus amieti* (African volcano frog)	23/2418.8253	Alpha helix: 78.26% Extended strand: 8.7% Beta turn: 13.04%		Stimulated enhanced production of pro-inflammatory interleukin IL-8 by oral fibroblasts	McLean et al. (2014)
Esculentin 2CHa		GFSSIFRGVAKFASKGLGKDLAKLGVDLVACKISKQC	*Lithobates chiricahuensis* (Chiricahua leopard frog)	37/3843.6182	Alpha helix: 75.68% Extended strand: 8.11% Beta turn: 8.11% Random coil: 8.11%		Enhanced the release and production of anti-inflammatory IL-10 by mouse lymphoid cells and also increase production of TNFα by peritoneal macrophages	Attoub et al. (2013)
Brevinin-2GU		GVIIDTLKGAAKTVAAELLRKAHCKLTNSC	*Hylarana guentheri*	30/3125.7520	Alpha helix: 76.67% Extended strand: 10% Beta turn: 6.67% Random coil: 6.67%		Downregulated the production of TNFα from Con A stimulated peripheral mononuclear cells and IFN-*γ* production in unstimulated cells	Popovic et al. (2012)
Temporin		HFLGKLVNLAKKIL	*R. draytonii*	14/1594.0203	Random coil: 100%		Upregulation of IL-10, IL-4, TGF-β from treated and untreated cells	Mangoni (2006)
Tigirin	IR	RVCSAIPLPICH		12/1308.6351	Random coil: 100%		Stimulated human peripheral blood mononuclear cells, as well as mouse peritoneal macrophages and splenocytes for increased the production of the anti-inflammatory cytokine IL-10 in both LPS-stimulated and unstimulated cells.	Ojo et al. (2013)
	IV	RICYAMWIPYPC	*Lithobates vaillanti*	12/1515.8875	Random coil: 100%			Conlon et al. (2009)
	IM	WCPPMIPLCSRF	*Xenopus muelleri*	12/1449.8282	Random coil: 100%			Ali et al. (2001)
Marine organisms								
Mytilus protein hydrolysate peptide		GVSLLQQFFL	*Mytilus coruscus* (Shell fish)	10/1151.3713	Random coil: 100%		Inhibited LPS-induced NO production in RAW264.7 macrophages	Kim et al. (2013)
Tilapapiscidin peptides	TP3	FIHHIIGGLFSVGKHIHSLIHGH	*Oreochromis niloticus* (Nile tilapa; cichild fish)	23/2557.0033	Alpha helix: 47.83% Extended strand: 21.74% Beta turn: 13.04% Random coil: 17.39%		Significantly increased the expression of several immune-related genes in muscle (IL-1β, IL-6, IL-8 TGF-β, and IκB) and decreased the expression of Toll-like receptor 5 (TLR5) to combat aquaculture bacterial pathogens	Lin et al. (2016)
	TP4	FIHHIIGGLFSAGKAIHRLIRRRRR		25/2981.6001	Alpha helix: 72% Extended strand: 12% Beta turn: 12% Random coil: 4%			
Phosvitin-derived peptide Pt5		SRMSKTATIIEPFRKFHKDRYLAHHSATKDTSSGSAAASFEQMQKQNRFLGNDIP	Zebra fish	55/6240.0122	Alpha helix: 50.91 Extended strand: 3.64% Beta turn: 7.27% Random coil: 38.18%		inhibits the expression of proinflammatory cytokine genes (IL-1β, IL-6, TNF-α, and IFN-γ) in the spleen and head kidneys of *A. hydrophila*-infected zebrafish, but increased the expression of anti-inflammatory cytokine genes (IL-10 and IL-14)	Ding et al. (2012)
Clavanin	A	VFQFLGKIIHHVGNFVHGFSHVF	*Styela clava* (TUNICATA)	23/2667.1150	Alpha helix: 86.96% Random coil: 13.04%	6C41	increased the level of IL-10, an anti-inflammatory cytokine, and decreased the levels of IL-12 and TNF-α, two pro-inflammatory cytokines that boost inflammation and may lead to excessive damage	Lee et al. (1997)
	MO	FLPIIVFQFLGKIIHHVGNFVHGFSHVF		28/3250.8868	Alpha helix: 67.86% Extended strand: 10.71% Random coil: 21.43%			Silva et al. (2016)
Birds								
β-defensins	AvBD2		Duck				Chemokine, CD4+ and CD8+ − T-cells and B-lymphocytes were chemotaxic toward peptide *invitro*. Downregulated C-type lecithin receptor in splenocytes	Soman et al. (2009)
	AvBD13		Chicken				Activated NF-κB cells, stimulated IL-12 and IFN-α production, and elevated CD80 and monocyte proliferation in murine PBMC cells,	Yang et al. (2010)
Cathelicidins	Cath-1 (Fowlicidin-1)	RVKRVWPLVIRTVIAGYNLYRAIKKK	Chicken	26/3141.8909	Alpha helix: 76.92% Beta turn: 3.85% Random coil: 19.23%	2AMN	Inhibited LPS-induced macrophage activation thereby inhibiting MCP-1,TNF α, IL-1α, and NO production in RAW264.7 mouse macrophages	Xiao et al. (2006b), Bommineni et al. (2007), and Xiao et al. (2006b)
	Cath-3 (Fowlicidin—3)	KRFWPLVPVAINTVAAGIN LYKAIRRK	Chicken	27/3095.7753	Alpha helix: 55.56% Extended strand: 11.11% Beta turn: 3.7% Random coil: 29.63%	2HFR		
	d-CATH	KRFWQLVPLAIKIYRAWKRR	Shaoxing ducks, *Anas platyrhynchos*	20/2629.2431	Alpha helix: 80.00% Beta turn: 10% Random coil: 10%		Anti-inflammatory effect: binds to LPS of bacterial cells	Feng et al. (2020)
Reptiles								
Cathelicidins	CWA		*Bungarus fascia (Branded krait snake)*				*Invitro* inhibited STAT and NF-κB pathway, thereby downregulating the production of pro-inflammatory cyotkines and enhanced the production of anti-inflammatory cytokines IL-4, IL-10 in *E. coli* K88-induced macrophages	Chen et al. (2018)
	CATH-4,5,6		*Alligator sinensis* (Alligator)				Inhibited the production of pro-inflammatory cytokines IL-6, IL-1β, TNFα, NO from LPS stimulated murine peritoneal macrophages	Chen et al. (2017)
	Cm-CATH2	RRSRFGRFFKKVRKQLGRVLRHSRITVGGRMRF	*Chelonia mydas* (green sea turtle)	33/4089.9366	Alpha helix: 60.61% Beta turn: 15.15% Random coil: 18.18%		Blocked TLR4/MD2 complex and the downstream signaling pathway activation, which increased the trafficking of neutrophils, macrophages, and monocytes to the infection site and reduced the generation of inflammatory cytokines caused by LPS.	Qiao et al. (2019)
	Hc-CATH	KFFKRLLKSVRRAVKKFRKKPRLIGLSTLL	*Hydrophis cyanocinctus (sea snake)*	30/3628.5931	Alpha helix: 60% Extended strand: 16.67% Random coil: 23.33%		Exhibited anti-inflammatory activity by downregulating the LPS-induced NO and pro-inflammatory cytokines such as TNF-α, IL-1β, and IL-6 production	Wei et al. (2015)

### 3.1 Host defence peptides from mammals: humans

Defensins, Cathelicidins, and Histatins are three categories of peptides endowed with antimicrobial as well as immunomodulatory functions (Guryanova and Ovchinnikova, 2022). Defensins are coded by genes present on chromosome 8 and are made of 30 amino acid residues held together with 3 cysteine disulfide bonds (Bowdish et al., 2006). Based on the type of disulfide bond, defensins are further classified into alpha-defensins and beta-defensins. Both *α* and *β* defensins are constitutively synthesized by lymphocytes, neutrophils, and epithelial cells of the mucous membrane and skin (Jarczak et al., 2013).

*α*-defensins (xCxCRxCxExGxCxGxCCx) are 2 to 6 kDa micropeptides abundant in azurophilic granules present in neutrophils. To date, six distinct α-defensins have been identified, including HNP-1, HNP-2, HNP-3, and HNP-4 (Xu and Lu, 2020) and enteric α-defensins-HD5 and HD6 secreted by Paneth cells of the gastrointestinal tract (Bevins and Salzman, 2011). α-defensins released from necrotic neutrophils can inhibit cytokines (TNF*α*, IL-6, IL-8, and IL-1β) that are secreted from macrophages, demonstrating anti-inflammatory activities (Miles et al., 2009). Human *α*-defensins also stimulate pro-inflammatory cytokines (IFN-*γ*, TNF-α) secretions, thereby stimulating the macrophages to enhance the phagocytotic activity (Soehnlein et al., 2008; Chaly et al., 2000). HNP1 and HNP3 defensins were found to inhibit monocyte differentiation (Droin et al., 2010). Enteric defensins (HD5, HD6) play a critical role in enhancing innate and adaptive immunity. They bind to toll-like receptors via MAP kinase pathway to transmit signals for the transcription of immune response genes, thereby initiating inflammation, wound healing and angiogenesis (Foureau et al., 2010; Eckmann, 2004). Human α-defensins increase the expression of the pro-inflammatory cytokines TNF-α and IL-1 in human monocytes (Chaly et al., 2000).

*β*-Defensins cluster present on chromosome 8, are released from epithelial cells and shield mucosal membrane from microbial invasions (Schutte et al., 2002). They are promiscuous in nature and can bind or interact with many receptors. β-Defensins (hBD1, hBD2) are chemotactic for immature dendritic and memory T cells (CD4+) (Yang et al., 1999) whereas hBD3 and 4 are chemotactic to monocytes (Wu et al., 2003). When combined with lipoteichoic acid cancer therapy, these peptides via the TLR2/NF-B signaling cascade increase the production of the chemokines (CCL20, CCL22, and CXL8) and cytokines (IL-1, IL-6, and IL-12) in human prostate cancer cells (Kim et al., 2015). Recent studies on the mechanism of hBD3-induced proinflammatory cytokine secretion revealed that hbD3 through TLR1/2 pathway elevates IL-1, IL-6, and IL-8 in human monocytes (Funderburg et al., 2011). There is a dearth of literature concerning the *in vivo* activity of β-Defensins. To date, only hBD-3 showed immunosuppressive activity under an *in vivo* setup (Semple et al., 2010).

Cathelicidin LL-37 is an *α*-helical peptide with 37 amino acid residues and the only cathelicidin synthesized in the human body. This peptide can trigger the synthesis of cytokines IL-6, IL-8, IL-10 and CCL2 either individually or in concert with IL-1 (Yu et al., 2007). Additionally, cathelicidin LL-37 promotes *α*-defensin production, thereby intensifying the inflammatory process (Zheng et al., 2007).

### 3.2 Host defence peptides from plants

Although plants have a very complex immune system. The bioactive peptides isolated from wheat, rice, maize, and soybean, have long been valued for their ability to control infections. These peptides have also been intensively explored for their immunomodulatory activities (Pavlicevic et al., 2022). Cationic defensins rich in cysteine amino acids bind to receptors activating neutrophils and macrophages to enhance innate and adaptive immunity. PEP1 and LR13 from *Oryza sativa* L (Rice) exhibited anti-inflammatory activity in both *in vitro* and *in vivo* conditions. The peptides were able to increase CD4+ and CD8+, thereby enhancing anti-inflammatory cytokines (IL-4, IL-10) and suppressing secretion of proinflammatory cytokines (IL-17, IFN-*γ*) (Shapira et al., 2010). Cyclolinopeptides D and G from *Linum usitatissimum* have been identified as modulators of proinflammatory responses, associated with increased secretion of IL-1β and TNF-α, while reducing IL-10 secretion in macrophages (Morita et al., 1999; Matsumoto et al., 2001). The second type of HDPs are less homogenous cryptic peptides produced in plants in response to antigens. Through the stimulation of natural killer cells, they can impact innate immunity (Lyapina et al., 2019). The GmSubPep peptide isolated from soybean leaves and synthesized by the extracellular subtilisin-like protease, can bind to membrane receptors and initiate the MAPK signaling cascade (Pearce et al., 2010). Also, the tomato compound CAP-derived peptide 1 (CAPE1) modifies protein–protein interactions and increases the transcription of antioxidative defence genes (Chen et al., 2014).

### 3.3 Host defence peptides from amphibians: frogs

Frogs are the largest reservoir of AMPs, which play a significant role in their defense mechanism. The skin secretions of the Pipidae frog family, including the genera *Silurana*, *Xenopus*, *Hymenochirus*, and *Pseudhymenochirus* are a rich source of AMPs with potent antimicrobial, immunomodulatory and anticancer activity (Conlon and Mechkarska, 2014; Patocka et al., 2019). Frog HDPs are produced in high concentrations and stored in the skin’s granular glands which are released immediately in retaliation to stress or tissue damage. These naturally occurring peptides are typically 8 to 48 amino acids in length and lack any conserved regions that are necessary for their therapeutic or biological activity. Most of them are cationic with hydrophobic amino acids and have shown therapeutic activity on mammalian cell lines (Conlon and Mechkarska, 2014). Frenatin 2D and Plasticin-L1 isolated from Alytidae and Leptodactylidae family of frogs did not show any anti-microbial activity but were found to stimulate the release of proinflammatory cytokines TNF-*α*, IL-1β, IL-12 from macrophages of mouse (Conlon et al., 2013; Scorciapino et al., 2013). Also, Plasticin-L1 enhanced IL-6 production but had no impact on anti-inflammatory IL-10 secretion (Scorciapino et al., 2013). The Tigerinins family of short, cyclic, cationic peptides with α -amidated C-terminus (present only in a few peptides) were isolated from the Dicroglossidae, Ranidae, and Pipidae families, demonstrated anti-inflammatory activity without hemolytic or antibacterial activity (Pantic et al., 2017). In both LPS-stimulated and unstimulated cells, they discovered that they could promote the production of the anti-inflammatory cytokine IL-10 by macrophages, splenocytes, and blood mononuclear cells (Pantic et al., 2014). Furthermore, tigerinin-1 V increased IL-6 production in LPS-triggered macrophages in mice. Tigerinin-1 M and -1 V significantly decreased IFN production in mononuclear cells isolated from mouse spleen, but had no impact on IL-17 release (Pantic et al., 2014). Studies have demonstrated that a number of HDPs, including the African clawed frog *Xenopus laevis*’s Magainin 1 and 2, Caerulein precursor fragment (CPF-AM1), and peptide glycine leucine amide (PGLa-AM1) stimulate the release of the immunomodulatory molecule glucagon-like peptide 1 (GLP-1), which reduces the immune system’s response to infection (Ojo et al., 2013; Insuela and Carvalho, 2017). Additionally, structurally distinct frog skin peptides, such as Esculentin-2CHa, Alyteserin-2a, and Pseudohymenochirins-1Pb and -2 Pa exhibited antibacterial and immunostimulatory properties (Pantic et al., 2017). However, no frog peptides have yet been used in clinical use as anti-infective or anti-inflammatory medicines. Further studies are underway, to understand HDPs interactions with immune cells and their impact on signaling pathways.

### 3.4 Host defence peptides from marine organisms

Peptides from marine organisms including fish, oyster, red algae, and mollusk demonstrate enhanced innate and adaptive immunity in host organisms. For example, Phosvitin-derived peptide Pt5 from *Danio rerio* increased the longevity rate of zebrafish infected with *Aeromonas hydrophila* by decreasing the expression of IL-1, IL-6, TNF-*α*, and IFN-*γ* secretions, while increasing the expression of IL-10 and IL-14 in spleen and kidney (Ding et al., 2012). In mice, Clavanin A and Clavanin-MO from *Styela clava* (Tunicate) altered cytokine synthesis by suppressing IL-12 and TNF-*α* and enhancing IL-1 (Lee et al., 1997; Silva et al., 2016). Shellfish Mytilus protein hydrolysate inhibited lipopolysaccharide stimulated nitrous oxide production in RAW 264.7 macrophages (Kim et al., 2013). Shark-derived protein hydrolysate (PeptiBal™) on oral administration enhanced intestinal cytokines (IL-6 and TNF-α) and immunoglobulin IgA production thereby leading to increase in TGF-*β* and IL-10. Thus indirectly decreasing the *E.coli* infection induced inflammation in the gut (Mallet et al., 2014). Although many HDPs from marine organisms were studied and formulated with biological enzymes and most of the peptides were tested only on animals. Clinical studies on humans still need to be conducted.

### 3.5 Host defence peptides from reptiles: snakes, crocodiles, lizards, turtles

*In-silico* analysis of reptile genomes (turtles, tortoise, snakes, lizards, crocodiles) was carried out to predict defensins and cathelicidins like peptides. In a lizard genome (*Anole carolinensis* or green anole) 32 β-defensin-like genes have been identified (Dalla Valle et al., 2012). First, *in vivo* role of β-defensins in wound healing and regeneration of lost tail was identified in the *Anole* lizard (Alibardi, 2013; Alibardi, 2014). Most of the β-defensin-like peptides found in lizards and snakes were expressed in heterophilic, azurophilic, and basophilic granulocytes whereas β-defensin (TBD-1) from turtles was found in leukocytes (Stegemann et al., 2009). However, no *α*-defensins have been identified. The second class of HDPs found in reptiles are cathelicidin-like peptides. Blast analysis with human cathelicidin revealed high similarity with cathelicidin-like peptides found in pit snakes, eastern brown snakes and elapid snakes (van Hoek, 2014; Zhao et al., 2008; Schmidt et al., 1992). Cathelicidin-like peptide genes have been also identified in Cobra king snake, *Anole* lizard, turtles, and crocodiles (van Hoek, 2014). However, no *in vitro* or *in vivo* studies were conducted to understand their immunomodulatory activity.

### 3.6 Host defence peptides from avians-birds

Like reptiles, only β-defensins were identified in birds. 14 chicken β-defensins cluster was identified on chromosome 3 (Hellgren and Ekblom, 2010) and three chicken Cathelicidin gene clusters were identified at a proximal end of chromosome 2 (Xiao et al., 2006a). The first avian defensin was isolated from leukocytes followed by the respiratory tract (AvBD 1, 2, 6, 10), reproductive system (testis- AvBD 1, 2, 4, 6, 10; ovary and oviduct AvBD 6, 10, 12) and spleen (AvBD 13). *In vivo* studies showed that duck AvBD2 has chemotactic activity toward CD4+, CD8+ T-cells and B-lymphocytes. This peptide was found to induce IFN-*γ* and IL-12 in mouse monocytes and enhanced CD3+, CD4+ and CD8+ T-cell proliferation (Cuperus et al., 2013). Cathelicidin especially Cath 1, 2, 3 are expressed in many tissues including lungs, tonsils, bone marrow, gastrointestinal tract, respiratory tract and lymphoid organs (Achanta et al., 2012). Cath 1 and 2 (fowlicidins) were found to inhibit the production of IL-1*α*, nitrous oxide, TNF-α and MCM-1 in mouse macrophages. They also inhibited lipopolysaccharide- induced macrophage activation (Bommineni et al., 2007; Xiao et al., 2006a; Xiao et al., 2006b).

## 4 The interaction between immunomodulatory HDPs and disease outcomes

Many of the diseases or disorders are associated with the immune system. As discussed in previous sections, HDPs directly or indirectly modulate immune cell secretions and release. These peptides play a critical role in disease progression and recovery. For example, Cathelicidin LL-37 was found to enhance increased uptake of CpG-oligonucleotide ligand by immune cells (CD4^+^ and CD8^+^ cells, B cells, neutrophils and macrophages), thereby enhancing the immunostimulatory and anti-tumor activity in ovarian cancer (Chuang et al., 2009). Human keratinocytes treated with Esculentin 1a (1–21), isolated from frog *Rana esculenta,* had enhanced STAT3 phosphorylation, thereby stimulating the transcription of downstream genes involved in wound healing (Di Grazia et al., 2015). By significantly altering the bovine neutrophil host defense peptide bactenecin, a small synthetic peptide known as innate defense regulator (IDR-)1018 was developed. This peptide acts as an immunoregulator, capable of suppressing the pro-inflammatory response by enhancing the production of selective chemokines and promoting cellular differentiation. It was found to enhance wound healing, anti-biofilm activity, cystic fibrosis and treatment of inflammatory diseases (neuronal damage and cerebral malaria) (Mansour et al., 2015). In both Type I and Type II diabetic Miletus, dysregulation in the HDP synthesis enhanced IFN-α synthesis leading to the progression of the disease (Sun et al., 2015). Also, low concentrations of HDPs were observed to enhance the pro-inflammatory responses thereby leading to multiple autoimmune disorders including rheumatoid arthritis, psoriasis, and systemic lupus erythematosus (SLE) (Kahlenberg and Kaplan, 2013).

## 5 The role of host defense peptides on immunomodulation in infectious disease management

Antimicrobial peptides are produced as key modulators of the innate immune system from various prokaryotic and eukaryotic organisms. Nowadays, with the alarming rise in infectious diseases, and bacterial resistance to traditional antibiotics, researchers are more inclined toward antimicrobial peptide-based treatment (Xuan et al., 2023). As we have previously discussed in the above sections, most of the AMPs from reptiles, amphibians and plants are studied on animal models and very less on human disease models. Among the very few peptides that have been studied, LL-37, a human cathelicidin, exhibits immunomodulatory activity as well as antimicrobial activity against *E. coli* and *Staphylococcus aureus* (Bhattacharjya et al., 2024). LL-37 promotes dendritic cell function contributing to efficient antigen presentation and activation of T cells in response to bacterial infections. Histatins, peptides obtained from human saliva, possess anti-bacterial and anti-fungal activity, by interfering with biofilm formation as well as activating host immune response (Kavanagh and Dowd, 2004). Protegrin-1, from porcine neutrophils, have immunosuppressive effect in chronic inflammatory diseases like sepsis (Javed et al., 2024). To tackle the SARS-CoV-2 pandemic, researchers developed models to incorporate defensins with T-cell and B-cell epitopes for developing vaccines against SARS-CoV-2. They also found that the binding of spike, nucleocapsid and membrane proteins with hBD-2 and hBD-3 escalates the immunogenic properties of the vaccine (Rahmani et al., 2022; Kumar et al., 2021; Guryanova and Ovchinnikova, 2022).

## 6 Prospects of peptides as therapeutics

Immunomodulatory host defense peptides affect a wide range of immune cells, including T-cells, B-cells, non-killer cells, macrophages, monocytes, CD4+ and CD8+ T cells. These peptides mainly act as ligands binding to Toll-like receptors transmitting signals via MAPK or TLR1/2 pathways enhancing activation of macrophages, stimulation of phagocytosis, an increase in leukocytes, increased production of immunoglobulins, and regulation of cytokines secretions, thereby modulating the innate and adaptive immunity in the host organisms. Due to these immunomodulatory activities, HDPs could be considered potent alternatives to antibiotics in the control of infections.

Clinical trials for many of these HDPs and their synthetic analogs are currently in various stages. Brilacidin, a synthetic peptide, has been effectively evaluated in phase II clinical trials for the treatment of acute bacterial skin infections. It has also been demonstrated to have antiviral activity against the SARS-CoV-2 virus (Hu et al., 2022). The Phase III clinical trials for pexiganan (MSI-78), an analog of magainin obtained from the African clawed frog *Xenopus laevis*, as a topical cream for diabetic foot ulcer treatment have been completed (Gomes et al., 2020). IDR-1 (Bactenecin), a synthetic peptide, is currently in phase I clinical trials to control inflammation, bacterial infection, and sepsis (Price et al., 2019). Some of the examples of synthetic peptides with immunomodulatory activity in phase II and III clinical trials were given in Table 2.

**Table 2:** List of immunomodulatory peptides under clinical trials.

Name of the peptide	Derived from	Developed by	Immunomodulatory activity	Clinical trial phase	References
EA230	Beta-chain of human gonadotropin	Exponential biotherapeutics	Upregulation of pro-inflammatory cytokines and neutrophil efflux	Phase II	van Groenendael et al. (2019)
CZEN-002	α-melanocyte-stimulating hormone	Zengen	Downregulation of TNF-α production	Phase II	Duncan and O’Neil (2013) and Fjell et al. (2012)
Delmitide (RDP 58)	HLA class I.	Genzyme	inhibition pro-inflammatory cytokines synthesis	Phase II	Travis et al. (2005)
Ghrelin	Host defense peptide (endogenous)	Royal Papworth Hospital (Cambridge, UK)	treatment of airway inflammation, chronic respiratory and lung infection	Phase II	Min et al. (2012) and Mookherjee et al. (2012)
Dusquetide (SGX942)	-	Soligenix	Modulation of innate immunity	Phase III	Kudrimoti et al. (2016)

However, the development of these naturally occurring or synthetic analogs as therapeutics is quite challenging and limited. The cost of producing synthetic peptides is approximately 50 to 400 USD per gram (Afacan et al., 2012). Some of the peptides in clinical trials were found to stimulate histamine production from mast cells, which can be toxic to host cells (Izumiya et al., 1981). The third major concern is their instability. Studies show simple HDPs without disulfide bonds are highly susceptible to proteolytic cleavage by host cell proteases (Kim et al., 2014).

## 7 Conclusion

The present review summarizes antimicrobial peptides as immunomodulatory agents. Most of the studies have shown that these peptides are effective in alleviating innate and adaptive immunity. These peptides are considered alternative therapeutics for the treatment of microbial infections, wound healing, inflammation control, diabetic care, cancer and auto-immune diseases. However, most of the studies are animal-based studies and not involving human patients. Only a few of them are in clinical trials for further commercial therapeutic applications. To comprehend their structural complexity, ligand-receptor interactions, and mechanism of action, more research is required. Furthermore, the safety and biocompatibility of host defense peptides must be explored to be developed as potent therapeutic agents.

## References

[ref1] Achanta M. Sunkara L. T. Dai G. Bommineni Y. R. Jiang W. Zhang G. (2012). Tissue expression and developmental regulation of chicken cathelicidin antimicrobial peptides. J. Anim. Sci. Biotechnol. 3:15. doi: 10.1186/2049-1891-3-15 22958518 PMC3436658

[ref2] Afacan J. Yeung A. T. Y. Pena O. M. Hancock R. E. W. (2012). Therapeutic potential of host defense peptides in antibiotic-resistant infections. Curr. Pharm. Des. 18, 807–819. doi: 10.2174/138161212799277617, PMID: 22236127

[ref3] Ali M. F. Soto A. Knoop F. C. Conlon J. M. (2001). Antimicrobial peptides isolated from skin secretions of the diploid frog, *Xenopus tropicalis* (Pipidae). Biochim. Biophys. Acta Protein Struct. Mol. Enzymol. 1550, 81–89. doi: 10.1016/S0167-4838(01)00272-2 11738090

[ref4] Alibardi L. (2013). Ultrastructural immunolocalization of beta-defensin-27 in granulocytes of the dermis and wound epidermis of lizard suggests they contribute to the anti-microbial skin barrier. Anat. Cell Biol. 46:246. doi: 10.5115/acb.2013.46.4.246 24386597 PMC3875842

[ref5] Alibardi L. (2014). Histochemical, biochemical and cell biological aspects of tail regeneration in lizard, an amniote model for studies on tissue regeneration. Prog. Histochem. Cytochem. 48, 143–244. doi: 10.1016/j.proghi.2013.12.001 24387878

[ref6] Allaker R. P. (2008). Host defence peptides—A bridge between the innate and adaptive immune responses. Trans. R. Soc. Trop. Med. Hyg. 102, 3–4. doi: 10.1016/j.trstmh.2007.07.005 17727907

[ref7] Attoub S. Mechkarska M. Sonnevend A. Radosavljevic G. Jovanovic I. Lukic M. L.. (2013). Esculentin-2CHa: A host-defense peptide with differential cytotoxicity against bacteria, erythrocytes and tumor cells. Peptides 39, 95–102. doi: 10.1016/j.peptides.2012.11.004 23159562

[ref8] Barlow P. G. Beaumont P. E. Cosseau C. Mackellar A. Wilkinson T. S. Hancock R. E. W.. (2010). The human cathelicidin LL-37 preferentially promotes apoptosis of infected airway epithelium. Am. J. Respir. Cell Mol. Biol. 43, 692–702. doi: 10.1165/rcmb.2009-0250OC 20097832 PMC2993089

[ref9] Bevins C. L. Salzman N. H. (2011). Paneth cells, antimicrobial peptides and maintenance of intestinal homeostasis. Nat. Rev. Microbiol. 9, 356–368. doi: 10.1038/nrmicro2546 21423246

[ref10] Bhattacharjya S. Zhang Z. Ramamoorthy A. (2024). LL-37: structures, antimicrobial activity, and influence on amyloid-related diseases. Biomol. Ther. 14:320. doi: 10.3390/biom14030320 PMC10968335 38540740

[ref11] Bommineni Y. R. Dai H. Gong Y.-X. Soulages J. L. Fernando S. C. DeSilva U.. (2007). Fowlicidin-3 is an α-helical cationic host defense peptide with potent antibacterial and lipopolysaccharide-neutralizing activities: structure and functions of fowlicidin-3. FEBS J. 274, 418–428. doi: 10.1111/j.1742-4658.2006.05589.x 17229147

[ref12] Bowdish D. M. E. Davidson D. J. Hancock R. E. W. (2006). “Immunomodulatory properties of Defensins and Cathelicidins” in Antimicrobial peptides and human disease. ed. Shafer W. M., vol. 306 (Heidelberg: Springer), 27–66. 10.1007/3-540-29916-5_2 PMC7121507 16909917

[ref13] Brook M. Tomlinson G. H. Miles K. Smith R. W. P. Rossi A. G. Hiemstra P. S.. (2016). Neutrophil-derived alpha defensins control inflammation by inhibiting macrophage mRNA translation. Proc. Natl. Acad. Sci. 113, 4350–4355. doi: 10.1073/pnas.1601831113, PMID: 27044108 PMC4843457

[ref14] Chaly Y. V. Paleolog E. M. Kolesnikova T. S. Tikhonov I. I. Petratchenko E. V. Voitenok N. N. (2000). Neutrophil alpha-defensin human neutrophil peptide modulates cytokine production in human monocytes and adhesion molecule expression in endothelial cells. Eur. Cytokine Netw. 11, 257–266. 10903805

[ref15] Chen Y. Cai S. Qiao X. Wu M. Guo Z. Wang R.. (2017). As-CATH1–6, novel cathelicidins with potent antimicrobial and immunomodulatory properties from *Alligator sinensis*, play pivotal roles in host antimicrobial immune responses. Biochem. J. 474, 2861–2885. doi: 10.1042/BCJ20170334 28798159

[ref16] Chen Y.-L. Lee C.-Y. Cheng K.-T. Chang W.-H. Huang R.-N. Nam H. G.. (2014). Quantitative Peptidomics study reveals that a wound-induced peptide from PR-1 regulates immune signaling in tomato. Plant Cell 26, 4135–4148. doi: 10.1105/tpc.114.131185 25361956 PMC4247587

[ref17] Chen S. Lu Z. Wang F. Wang Y. (2018). Cathelicidin-WA polarizes *E. coli* K88-induced M1 macrophage to M2-like macrophage in RAW264.7 cells. Int. Immunopharmacol. 54, 52–59. doi: 10.1016/j.intimp.2017.10.013 29101873

[ref18] Chen X. Takai T. Xie Y. Niyonsaba F. Okumura K. Ogawa H. (2013). Human antimicrobial peptide LL-37 modulates proinflammatory responses induced by cytokine milieus and double-stranded RNA in human keratinocytes. Biochem. Biophys. Res. Commun. 433, 532–537. doi: 10.1016/j.bbrc.2013.03.024 23524263

[ref19] Choi K.-Y. Mookherjee N. (2012). Multiple immune-modulatory functions of cathelicidin host defense peptides. Front. Immunol. 3:149. doi: 10.3389/fimmu.2012.00149 22701455 PMC3371594

[ref20] Chuang C.-M. Monie A. Wu A. Mao C.-P. Hung C.-F. (2009). Treatment with LL-37 peptide enhances antitumor effects induced by CpG oligodeoxynucleotides against ovarian cancer. Hum. Gene Ther. 20, 303–313. doi: 10.1089/hum.2008.124 19272013 PMC2855250

[ref21] Conlon J. M. (2015). Host-defense peptides of the skin with therapeutic potential: from hagfish to human. Peptides 67, 29–38. doi: 10.1016/j.peptides.2015.03.005 25794853

[ref22] Conlon J. M. Mechkarska M. (2014). Host-defense peptides with therapeutic potential from skin secretions of frogs from the family Pipidae. Pharmaceuticals 7, 58–77. doi: 10.3390/ph7010058 24434793 PMC3915195

[ref23] Conlon J. M. Mechkarska M. Pantic J. M. Lukic M. L. Coquet L. Leprince J.. (2013). An immunomodulatory peptide related to Frenatin 2 from skin secretions of the *Tyrrhenian* painted frog *Discoglossus sardus* (Alytidae). Peptides 40, 65–71. doi: 10.1016/j.peptides.2012.12.012 23262358

[ref24] Conlon J. M. Raza H. Coquet L. Jouenne T. Leprince J. Vaudry H.. (2009). Purification of peptides with differential cytolytic activities from the skin secretions of the central American frog, *Lithobates vaillanti* (Ranidae). Comparat. Biochem. Physiol. Part C 150, 150–154. doi: 10.1016/j.cbpc.2009.04.003 19379837

[ref25] Cuperus T. Coorens M. van Dijk A. Haagsman H. P. (2013). Avian host defense peptides. Dev. Compar. Immunol. 41, 352–369. doi: 10.1016/j.dci.2013.04.019 23644014

[ref26] Dalla Valle L. Benato F. Maistro S. Quinzani S. Alibardi L. (2012). Bioinformatic and molecular characterization of beta-defensins-like peptides isolated from the green lizard *Anolis carolinensis*. Dev. Compar. Immunol. 36, 222–229. doi: 10.1016/j.dci.2011.05.004 21663758

[ref27] Davidson D. J. Currie A. J. Reid G. S. D. Bowdish D. M. E. MacDonald K. L. Ma R. C.. (2004). The cationic antimicrobial peptide LL-37 modulates dendritic cell differentiation and dendritic cell-induced T cell polarization. J. Immunol. 172, 1146–1156. doi: 10.4049/jimmunol.172.2.1146 14707090

[ref28] de la Fuente-Núñez C. Reffuveille F. Haney E. F. Straus S. K. Hancock R. E. W. (2014). Broad-Spectrum anti-biofilm peptide that targets a cellular stress response. PLoS Pathog. 10:e1004152. doi: 10.1371/journal.ppat.1004152 24852171 PMC4031209

[ref29] Di Grazia A. Cappiello F. Imanishi A. Mastrofrancesco A. Picardo M. Paus R.. (2015). The frog skin-derived antimicrobial peptide Esculentin-1a(1-21)NH2 promotes the migration of human HaCaT keratinocytes in an EGF receptor-dependent manner: A novel promoter of human skin wound healing? PLoS One 10:e0128663. doi: 10.1371/journal.pone.0128663 26068861 PMC4466536

[ref30] Ding Y. Liu X. Bu L. Li H. Zhang S. (2012). Antimicrobial-immunomodulatory activities of zebrafish phosvitin-derived peptide Pt5. Peptides 37, 309–313. doi: 10.1016/j.peptides.2012.07.014 22841856

[ref31] Dlozi P. N. Gladchuk A. Crutchley R. D. Keuler N. Coetzee R. Dube A. (2022). Cathelicidins and defensins antimicrobial host defense peptides in the treatment of TB and HIV: Pharmacogenomic and nanomedicine approaches towards improved therapeutic outcomes. Biomed. Pharmacother. 151:113189. doi: 10.1016/j.biopha.2022.113189 35676789 PMC9209695

[ref32] Droin N. Jacquel A. Hendra J.-B. Racoeur C. Truntzer C. Pecqueur D.. (2010). Alpha-defensins secreted by dysplastic granulocytes inhibit the differentiation of monocytes in chronic myelomonocytic leukemia. Blood 115, 78–88. doi: 10.1182/blood-2009-05-224352 19864642

[ref33] Duncan V. M. S. O’Neil D. A. (2013). Commercialization of antifungal peptides. Fungal Biol. Rev. 26, 156–165. doi: 10.1016/j.fbr.2012.11.001

[ref34] Eckmann L. (2004). Innate immunity and mucosal bacterial interactions in the intestine. Curr. Opin. Gastroenterol. 20, 82–88. doi: 10.1097/00001574-200403000-00006 15703626

[ref35] Feng X. Jin S. Wang M. Pang Q. Liu C. Liu R.. (2020). The critical role of tryptophan in the antimicrobial activity and cell toxicity of the duck antimicrobial peptide DCATH. Front. Microbiol. 11:1146. doi: 10.3389/fmicb.2020.01146 32670215 PMC7326067

[ref9004] Fernandez de Caleya R. Gonzalez-Pascual B. García-Olmedo F. Carbonero P. (1972). Susceptibility of phytopathogenic bacteria to wheat purothionins in vitro. Appl Microbiol. 23, 998–1000. doi: 10.1128/am.23.5.998-1000.1972 5031563 PMC380489

[ref36] Fjell C. D. Hiss J. A. Hancock R. E. W. Schneider G. (2012). Designing antimicrobial peptides: form follows function. Nat. Rev. Drug Discov. 11, 37–51. doi: 10.1038/nrd3591 22173434

[ref37] Foureau D. M. Mielcarz D. W. Menard L. C. Schulthess J. Werts C. Vasseur V.. (2010). TLR9-dependent induction of intestinal α-Defensins by *Toxoplasma gondii*. J. Immunol. 184, 7022–7029. doi: 10.4049/jimmunol.0901642 20488791

[ref38] Funderburg N. T. Jadlowsky J. K. Lederman M. M. Feng Z. Weinberg A. Sieg S. F. (2011). The toll-like receptor 1/2 agonists Pam3CSK4 and human β-defensin-3 differentially induce interleukin-10 and nuclear factor-κB signalling patterns in human monocytes: differential activation of APCs by TLR1/2 ligands. Immunology 134, 151–160. doi: 10.1111/j.1365-2567.2011.03475.x 21896010 PMC3194223

[ref39] Gilding E. K. Jackson M. A. Poth A. G. Henriques S. T. Prentis P. J. Mahatmanto T.. (2016). Gene coevolution and regulation lock cyclic plant defence peptides to their targets. New Phytol. 210, 717–730. doi: 10.1111/nph.13789 26668107

[ref40] Gomes D. Santos R. Reis S. Carvalho S. Rego P. Tavares L.. (2020). Pexiganan in combination with Nisin to control Polymicrobial diabetic foot infections. Antibiotics 9:128. doi: 10.3390/antibiotics9030128, PMID: 32244862 PMC7148459

[ref41] Guryanova S. V. Ovchinnikova T. V. (2022). Immunomodulatory and allergenic properties of antimicrobial peptides. Int. J. Mol. Sci. 23:2499. doi: 10.3390/ijms23052499 35269641 PMC8910669

[ref42] Hancock R. E. W. Haney E. F. Gill E. E. (2016). The immunology of host defence peptides: beyond antimicrobial activity. Nat. Rev. Immunol. 16, 321–334. doi: 10.1038/nri.2016.29 27087664

[ref43] Heinbockel L. Weindl G. Correa W. Brandenburg J. Reiling N. Wiesmüller K.-H.. (2021). Anti-infective and anti-inflammatory mode of action of peptide 19-2.5. Int. J. Mol. Sci. 22:1465. doi: 10.3390/ijms22031465 33540553 PMC7867136

[ref44] Hellgren O. Ekblom R. (2010). Evolution of a cluster of innate immune genes (β-defensins) along the ancestral lines of chicken and zebra finch. Immunome Res. 6:3. doi: 10.1186/1745-7580-6-3 20359324 PMC3161384

[ref45] Hemshekhar M. Choi K.-Y. G. Mookherjee N. (2018). Host defense peptide LL-37-mediated chemoattractant properties, but not anti-inflammatory cytokine IL-1RA production, is selectively controlled by Cdc42 rho GTPase via G protein-coupled receptors and JNK mitogen-activated protein kinase. Front. Immunol. 9:1871. doi: 10.3389/fimmu.2018.01871 30158931 PMC6104452

[ref46] Hoover D. M. Chertov O. Lubkowski J. (2001). The structure of human β-Defensin-1. J. Biol. Chem. 276, 39021–39026. doi: 10.1074/jbc.M103830200 11486002

[ref47] Hu Y. Jo H. DeGrado W. F. Wang J. (2022). Brilacidin, a COVID-19 drug candidate, demonstrates broad-spectrum antiviral activity against human coronaviruses OC43, 229E, and NL63 through targeting both the virus and the host cell. J. Med. Virol. 94, 2188–2200. doi: 10.1002/jmv.27616 35080027 PMC8930451

[ref9001] Huan Y. Kong Q. Mou H. Yi H. (2020). Antimicrobial Peptides: Classification, Design, Application and Research Progress in Multiple Fields. Front Microbiol. 11:582779. Published 2020 Oct 16. doi: 10.3389/fmicb.2020.582779 33178164 PMC7596191

[ref48] Insuela D. B. R. Carvalho V. F. (2017). Glucagon and glucagon-like peptide-1 as novel anti-inflammatory and immunomodulatory compounds. Eur. J. Pharmacol. 812, 64–72. doi: 10.1016/j.ejphar.2017.07.015 28688914

[ref49] Izumiya N. Kato T. Waki M. (1981). Synthesis of biologically active cyclic peptides. Biopolymers 20, 1785–1791. doi: 10.1002/bip.1981.360200903 7306667

[ref50] Jarczak J. Kościuczuk E. M. Lisowski P. Strzałkowska N. Jóźwik A. Horbańczuk J.. (2013). Defensins: natural component of human innate immunity. Hum. Immunol. 74, 1069–1079. doi: 10.1016/j.humimm.2013.05.008 23756165

[ref51] Javed A. Oedairadjsingh T. Ludwig I. S. Wood T. M. Martin N. I. Broere F.. (2024). Antimicrobial and immunomodulatory activities of porcine cathelicidin Protegrin-1. Mol. Immunol. 173, 100–109. doi: 10.1016/j.molimm.2024.07.011 39094445

[ref52] Jhong J.-H. Yao L. Pang Y. Li Z. Chung C.-R. Wang R.. (2022). dbAMP 2.0: updated resource for antimicrobial peptides with an enhanced scanning method for genomic and proteomic data. Nucleic Acids Res. 50, D460–D470. doi: 10.1093/nar/gkab1080 34850155 PMC8690246

[ref53] Kahlenberg J. M. Kaplan M. J. (2013). Little peptide, big effects: the role of LL-37 in inflammation and autoimmune disease. J. Immunol. 191, 4895–4901. doi: 10.4049/jimmunol.1302005 24185823 PMC3836506

[ref54] Kavanagh K. Dowd S. (2004). Histatins: antimicrobial peptides with therapeutic potential. J. Pharm. Pharmacol. 56, 285–289. doi: 10.1211/0022357022971 15025852

[ref55] Kim H. Jang J. H. Kim S. C. Cho J. H. (2014). De novo generation of short antimicrobial peptides with enhanced stability and cell specificity. J. Antimicrob. Chemother. 69, 121–132. doi: 10.1093/jac/dkt322 23946320

[ref56] Kim E.-K. Kim Y.-S. Hwang J.-W. Kang S. H. Choi D.-K. Lee K.-H.. (2013). Purification of a novel nitric oxide inhibitory peptide derived from enzymatic hydrolysates of *Mytilus coruscus*. Fish Shellfish Immunol. 34, 1416–1420. doi: 10.1016/j.fsi.2013.02.023 23500953

[ref57] Kim J. H. Kim K.-H. Kim H. J. Lee J. Myung S. C. (2015). Expression of Beta-Defensin 131 promotes an innate immune response in human prostate epithelial cells. PLoS One 10:e0144776. doi: 10.1371/journal.pone.0144776 26649771 PMC4674080

[ref58] Koeninger L. Armbruster N. S. Brinch K. S. Kjaerulf S. Andersen B. Langnau C.. (2020). Human β-Defensin 2 mediated immune modulation as treatment for experimental colitis. Front. Immunol. 11:93. doi: 10.3389/fimmu.2020.00093 32076420 PMC7006816

[ref59] Kudrimoti M. Curtis A. Azawi S. Worden F. Katz S. Adkins D.. (2016). Dusquetide: A novel innate defense regulator demonstrating a significant and consistent reduction in the duration of oral mucositis in preclinical data and a randomized, placebo-controlled phase 2a clinical study. J. Biotechnol. 239, 115–125. doi: 10.1016/j.jbiotec.2016.10.010 27746305

[ref9005] Kühnle N. Dederer V. Lemberg M. K. (2019). Intramembrane proteolysis at a glance: from signalling to protein degradation. J Cell Sci. 132:jcs217745. Published 2019 Aug 15. doi: 10.1242/jcs.217745 31416853

[ref60] Kumar V. Kancharla S. Kolli P. Jena M. (2021). Reverse vaccinology approach towards the *in-silico* multiepitope vaccine development against SARS-CoV-2. F1000Research 10:44. doi: 10.12688/f1000research.36371.1 33841800 PMC8009247

[ref61] Lammers K. M. Khandelwal S. Chaudhry F. Kryszak D. Puppa E. L. Casolaro V.. (2011). Identification of a novel immunomodulatory gliadin peptide that causes interleukin-8 release in a chemokine receptor CXCR3-dependent manner only in patients with coeliac disease: gliadin-induced IL-8 is CXCR3-dependent only in CD patients. Immunology 132, 432–440. doi: 10.1111/j.1365-2567.2010.03378.x 21091908 PMC3044909

[ref62] Lee I. H. Cho Y. Lehrer R. I. (1997). Effects of pH and salinity on the antimicrobial properties of clavanins. Infect. Immun. 65, 2898–2903. doi: 10.1128/iai.65.7.2898-2903.1997 9199465 PMC175407

[ref63] Lehrer R. I. Lu W. (2012). α-Defensins in human innate immunity: α-Defensins. Immunol. Rev. 245, 84–112. doi: 10.1111/j.1600-065X.2011.01082.x 22168415

[ref64] Lesiuk M. Paduszyńska M. Greber K. E. (2022). Synthetic antimicrobial immunomodulatory peptides: ongoing studies and clinical trials. Antibiotics 11:1062. doi: 10.3390/antibiotics11081062 36009931 PMC9405281

[ref65] Lin W.-C. Chang H.-Y. Chen J.-Y. (2016). Electrotransfer of the tilapia piscidin 3 and tilapia piscidin 4 genes into skeletal muscle enhances the antibacterial and immunomodulatory functions of *Oreochromis niloticus*. Fish Shellfish Immunol. 50, 200–209. doi: 10.1016/j.fsi.2016.01.034 26828260

[ref66] Liu P. Liao W. Qi X. Yu W. Wu J. (2020). Identification of immunomodulatory peptides from zein hydrolysates. Eur. Food Res. Technol. 246, 931–937. doi: 10.1007/s00217-020-03450-x

[ref67] Luo Y. Song Y. (2021). Mechanism of antimicrobial peptides: antimicrobial, anti-inflammatory and Antibiofilm activities. Int. J. Mol. Sci. 22:11401. doi: 10.3390/ijms222111401 34768832 PMC8584040

[ref9002] Luong H. X. Thanh T. T. Tran T. H. (2020). Antimicrobial peptides - Advances in development of therapeutic applications. Life Sci. 260:118407. doi: 10.1016/j.lfs.2020.118407 32931796 PMC7486823

[ref68] Lyapina I. Filippova A. Fesenko I. (2019). The role of peptide signals hidden in the structure of functional proteins in plant immune responses. Int. J. Mol. Sci. 20:4343. doi: 10.3390/ijms20184343 31491850 PMC6770897

[ref69] Mallet J.-F. Duarte J. Vinderola G. Anguenot R. Beaulieu M. Matar C. (2014). The immunopotentiating effects of shark-derived protein hydrolysate. Nutrition 30, 706–712. doi: 10.1016/j.nut.2013.10.025 24800670

[ref70] Mangoni M. L. (2006). Temporins, anti-infective peptides with expanding properties. Cell. Mol. Life Sci. 63, 1060–1069. doi: 10.1007/s00018-005-5536-y 16572270 PMC11136197

[ref71] Mansour S. C. de la Fuente-Núñez C. Hancock R. E. W. (2015). Peptide IDR-1018: modulating the immune system and targeting bacterial biofilms to treat antibiotic-resistant bacterial infections: immunomodulatory and anti-biofilm peptide. J. Pept. Sci. 21, 323–329. doi: 10.1002/psc.2708 25358509

[ref72] Matsumoto T. Shishido A. Morita H. Itokawa H. Takeya K. (2001). Cyclolinopeptides F–I, cyclic peptides from linseed. Phytochemistry 57, 251–260. doi: 10.1016/S0031-9422(00)00442-8 11382241

[ref73] McLean D. T. F. McCrudden M. T. C. Linden G. J. Irwin C. R. Conlon J. M. Lundy F. T. (2014). Antimicrobial and immunomodulatory properties of PGLa-AM1, CPF-AM1, and magainin-AM1: potent activity against oral pathogens. Regul. Pept. 194–195, 63–68. doi: 10.1016/j.regpep.2014.11.002 25447193

[ref74] Mechkarska M. Attoub S. Sulaiman S. Pantic J. Lukic M. L. Michael Conlon J. (2014). Anti-cancer, immunoregulatory, and antimicrobial activities of the frog skin host-defense peptides pseudhymenochirin-1Pb and pseudhymenochirin-2Pa. Regul. Pept. 194–195, 69–76. doi: 10.1016/j.regpep.2014.11.001 25447194

[ref75] Miles K. Clarke D. J. Lu W. Sibinska Z. Beaumont P. E. Davidson D. J.. (2009). Dying and necrotic neutrophils are anti-inflammatory secondary to the release of α-Defensins. J. Immunol. 183, 2122–2132. doi: 10.4049/jimmunol.0804187 19596979 PMC2948539

[ref76] Min C. Ohta K. Kajiya M. Zhu T. Sharma K. Shin J.. (2012). The antimicrobial activity of the appetite peptide hormone ghrelin. Peptides 36, 151–156. doi: 10.1016/j.peptides.2012.05.006 22634233 PMC3402649

[ref77] Mookherjee N. Anderson M. A. Haagsman H. P. Davidson D. J. (2020). Antimicrobial host defence peptides: functions and clinical potential. Nat. Rev. Drug Discov. 19, 311–332. doi: 10.1038/s41573-019-0058-8 32107480

[ref78] Mookherjee N. Chow L. N. Y. Hancock R. E. W. (2012). “Immunomodulatory cationic peptide therapeutics: A new paradigm in infection and immunity” in ACS symposium series. eds. Rajasekaran K. Cary J. W. Jaynes J. M. Montesinos E., vol. 1095 (Washington, DC: American Chemical Society), 1–19.

[ref79] Morita H. Shishido A. Matsumoto T. Itokawa H. Takeya K. (1999). Cyclolinopeptides B - E, new cyclic peptides from *Linum usitatissimum*. Tetrahedron 55, 967–976. doi: 10.1016/S0040-4020(98)01086-2

[ref80] Nell M. J. Tjabringa G. S. Wafelman A. R. Verrijk R. Hiemstra P. S. Drijfhout J. W.. (2006). Development of novel LL-37 derived antimicrobial peptides with LPS and LTA neutralizing and antimicrobial activities for therapeutic application. Peptides 27, 649–660. doi: 10.1016/j.peptides.2005.09.016 16274847

[ref81] Nguyen G. K. T. Zhang S. Nguyen N. T. K. Nguyen P. Q. T. Chiu M. S. Hardjojo A.. (2011). Discovery and characterization of novel Cyclotides originated from chimeric precursors consisting of Albumin-1 chain A and Cyclotide domains in the Fabaceae Family. J. Biol. Chem. 286, 24275–24287. doi: 10.1074/jbc.M111.229922 21596752 PMC3129208

[ref82] Nijnik A. Madera L. Ma S. Waldbrook M. Elliott M. R. Easton D. M.. (2010). Synthetic cationic peptide IDR-1002 provides protection against bacterial infections through chemokine induction and enhanced leukocyte recruitment. J. Immunol. 184, 2539–2550. doi: 10.4049/jimmunol.0901813 20107187

[ref83] Ojo O. O. Conlon J. M. Flatt P. R. Abdel-Wahab Y. H. A. (2013). Frog skin peptides (tigerinin-1R, magainin-AM1, −AM2, CPF-AM1, and PGla-AM1) stimulate secretion of glucagon-like peptide 1 (GLP-1) by GLUTag cells. Biochem. Biophys. Res. Commun. 431, 14–18. doi: 10.1016/j.bbrc.2012.12.116 23291176

[ref84] Overhage J. Campisano A. Bains M. Torfs E. C. W. Rehm B. H. A. Hancock R. E. W. (2008). Human host defense peptide LL-37 prevents bacterial biofilm formation. Infect. Immun. 76, 4176–4182. doi: 10.1128/IAI.00318-08 18591225 PMC2519444

[ref85] Pachón-Ibáñez M. E. Smani Y. Pachón J. Sánchez-Céspedes J. (2017). Perspectives for clinical use of engineered human host defense antimicrobial peptides. FEMS Microbiol. Rev. 41, 323–342. doi: 10.1093/femsre/fux012 28521337 PMC5435762

[ref86] Pantic J. M. Jovanovic I. P. Radosavljevic G. D. Arsenijevic N. N. Conlon J. M. Lukic M. L. (2017). The potential of frog skin-derived peptides for development into therapeutically-valuable immunomodulatory agents. Molecules 22:2071. doi: 10.3390/molecules22122071 29236056 PMC6150033

[ref87] Pantic J. M. Mechkarska M. Lukic M. L. Conlon J. M. (2014). Effects of tigerinin peptides on cytokine production by mouse peritoneal macrophages and spleen cells and by human peripheral blood mononuclear cells. Biochimie 101, 83–92. doi: 10.1016/j.biochi.2013.12.022 24412102

[ref9003] Pasupuleti M. Schmidtchen A. Malmsten M. (2012). Antimicrobial peptides: key components of the innate immune system. Crit Rev Biotechnol. 32, 143–171. doi: 10.3109/07388551.2011.594423 22074402

[ref88] Patocka J. Nepovimova E. Klimova B. Wu Q. Kuca K. (2019). Antimicrobial peptides: amphibian host defense peptides. Curr. Med. Chem. 26, 5924–5946. doi: 10.2174/0929867325666180713125314 30009702

[ref89] Pavlicevic M. Marmiroli N. Maestri E. (2022). Immunomodulatory peptides—A promising source for novel functional food production and drug discovery. Peptides 148:170696. doi: 10.1016/j.peptides.2021.170696 34856531

[ref90] Pearce G. Munske G. Yamaguchi Y. Ryan C. A. (2010). Structure–activity studies of GmSubPep, a soybean peptide defense signal derived from an extracellular protease. Peptides 31, 2159–2164. doi: 10.1016/j.peptides.2010.09.004 20833217

[ref91] Petkovic M. Mouritzen M. V. Mojsoska B. Jenssen H. (2021). Immunomodulatory properties of host Defence peptides in skin wound healing. Biomol. Ther. 11:952. doi: 10.3390/biom11070952 PMC8301823 34203393

[ref92] Popovic S. Urbán E. Lukic M. Conlon J. M. (2012). Peptides with antimicrobial and anti-inflammatory activities that have therapeutic potential for treatment of acne vulgaris. Peptides 34, 275–282. doi: 10.1016/j.peptides.2012.02.010 22374306

[ref93] Price R. L. Bugeon L. Mostowy S. Makendi C. Wren B. W. Williams H. D.. (2019). *In vitro* and *in vivo* properties of the bovine antimicrobial peptide, Bactenecin 5. PLoS One 14:e0210508. doi: 10.1371/journal.pone.0210508 30625198 PMC6326515

[ref94] Qiao X. Yang H. Gao J. Zhang F. Chu P. Yang Y.. (2019). Diversity, immunoregulatory action and structure-activity relationship of green sea turtle cathelicidins. Dev. Comparat. Immunol. 98, 189–204. doi: 10.1016/j.dci.2019.05.005 31121185

[ref95] Rahmani A. Baee M. Saleki K. Moradi S. Nouri H. R. (2022). Applying high throughput and comprehensive immunoinformatics approaches to design a trivalent subunit vaccine for induction of immune response against emerging human coronaviruses SARS-CoV, MERS-CoV and SARS-CoV-2. J. Biomol. Struct. Dyn. 40, 6097–6113. doi: 10.1080/07391102.2021.1876774 33509045 PMC7852294

[ref96] Rajasekaran G. Dinesh Kumar S. Nam J. Jeon D. Kim Y. Lee C. W.. (2019). Antimicrobial and anti-inflammatory activities of chemokine CXCL14-derived antimicrobial peptide and its analogs. Biomembranes 1861, 256–267. doi: 10.1016/j.bbamem.2018.06.016 29959905

[ref97] Rivas-Santiago B. Castañeda-Delgado J. E. Rivas Santiago C. E. Waldbrook M. González-Curiel I. León-Contreras J. C.. (2013). Ability of innate Defence regulator peptides IDR-1002, IDR-HH2 and IDR-1018 to protect against *Mycobacterium tuberculosis* infections in animal models. PLoS One 8:e59119. doi: 10.1371/journal.pone.0059119 23555622 PMC3605426

[ref98] Sandra Tjabringa G. Rabe K. F. Hiemstra P. S. (2005). The human cathelicidin LL-37: A multifunctional peptide involved in infection and inflammation in the lung. Pulm. Pharmacol. Ther. 18, 321–327. doi: 10.1016/j.pupt.2005.01.001 15939310

[ref99] Schmidt J. J. Weinstein S. A. Smith L. A. (1992). Molecular properties and structure-function relationships of lethal peptides from venom of Wagler’s pit viper, *Trimeresurus wagleri*. Toxicon 30, 1027–1036. doi: 10.1016/0041-0101(92)90047-9 1440639

[ref100] Schröder J.-M. Harder J. (1999). Human beta-defensin-2. Int. J. Biochem. Cell Biol. 31, 645–651. doi: 10.1016/S1357-2725(99)00013-8 10404637

[ref101] Schutte B. C. Mitros J. P. Bartlett J. A. Walters J. D. Jia H. P. Welsh M. J.. (2002). Discovery of five conserved β-defensin gene clusters using a computational search strategy. Proc. Natl. Acad. Sci. 99, 2129–2133. doi: 10.1073/pnas.042692699 11854508 PMC122330

[ref102] Scorciapino M. A. Manzo G. Rinaldi A. C. Sanna R. Casu M. Pantic J. M.. (2013). Conformational analysis of the frog skin peptide, Plasticin-L1, and its effects on production of Proinflammatory cytokines by macrophages. Biochemistry 52, 7231–7241. doi: 10.1021/bi4008287 24073891

[ref103] Semple F. Dorin J. R. (2012). β-Defensins: multifunctional modulators of infection, inflammation and more? J. Innate Immun. 4, 337–348. doi: 10.1159/000336619 22441423 PMC6784047

[ref104] Semple F. Webb S. Li H.-N. Patel H. B. Perretti M. Jackson I. J.. (2010). Human β-defensin 3 has immunosuppressive activity *in vitro* and *in vivo*: immunomodulation. Eur. J. Immunol. 40, 1073–1078. doi: 10.1002/eji.200940041 20104491 PMC2948537

[ref105] Serra A. Hemu X. Nguyen G. K. T. Nguyen N. T. K. Sze S. K. Tam J. P. (2016). A high-throughput peptidomic strategy to decipher the molecular diversity of cyclic cysteine-rich peptides. Sci. Rep. 6:23005. doi: 10.1038/srep23005 26965458 PMC4786859

[ref106] Shapira E. Brodsky B. Proscura E. Nyska A. Erlanger-Rosengarten A. Wormser U. (2010). Amelioration of experimental autoimmune encephalitis by novel peptides: involvement of T regulatory cells. J. Autoimmun. 35, 98–106. doi: 10.1016/j.jaut.2010.03.004 20434883

[ref107] Sharma M. Sharma S. Prasad R. Rajwanshi A. Sethi S. Samanta P.. (2011). Characterization of low molecular weight antimicrobial peptide from human female reproductive tract. Indian J. Med. Res. 134:679. doi: 10.4103/0971-5916.90996 22199108 PMC3249967

[ref108] Silva O. N. de la Fuente-Núñez C. Haney E. F. Fensterseifer I. C. M. Ribeiro S. M. Porto W. F.. (2016). An anti-infective synthetic peptide with dual antimicrobial and immunomodulatory activities. Sci. Rep. 6:35465. doi: 10.1038/srep35465 27804992 PMC5090204

[ref109] Soehnlein O. Kai-Larsen Y. Frithiof R. Sorensen O. E. Kenne E. Scharffetter-Kochanek K.. (2008). Neutrophil primary granule proteins HBP and HNP1–3 boost bacterial phagocytosis by human and murine macrophages. J. Clin. Invest. 118, 3491–3502. doi: 10.1172/JCI35740 18787642 PMC2532980

[ref110] Soman S. S. Nair S. Issac A. Arathy D. S. Niyas K. P. Anoop M.. (2009). Immunomodulation by duck defensin, Apl_AvBD2: *in vitro* dendritic cell immunoreceptor (DCIR) mRNA suppression, and B- and T-lymphocyte chemotaxis. Mol. Immunol. 46, 3070–3075. doi: 10.1016/j.molimm.2009.06.003 19577301

[ref111] Stegemann C. Kolobov A. Leonova Y. F. Knappe D. Shamova O. Ovchinnikova T. V.. (2009). Isolation, purification and de novo sequencing of TBD-1, the first beta-defensin from leukocytes of reptiles. Proteomics 9, 1364–1373. doi: 10.1002/pmic.200800569 19253295

[ref112] Sun J. Furio L. Mecheri R. van der Does A. M. Lundeberg E. Saveanu L.. (2015). Pancreatic β-cells limit autoimmune diabetes via an immunoregulatory antimicrobial peptide expressed under the influence of the gut microbiota. Immunity 43, 304–317. doi: 10.1016/j.immuni.2015.07.013 26253786

[ref113] Travis S. Yap L. M. Hawkey C. Warren B. Lazarov M. Fong T.. (2005). Rdp58 is a novel and potentially effective Oral therapy for ulcerative colitis. Inflamm. Bowel Dis. 11, 713–719. doi: 10.1097/01.MIB.0000172807.26748.16 16043985

[ref114] Valore E. V. Park C. H. Quayle A. J. Wiles K. R. McCray P. B. Ganz T. (1998). Human beta-defensin-1: an antimicrobial peptide of urogenital tissues. J. Clin. Invest. 101, 1633–1642. doi: 10.1172/JCI1861 9541493 PMC508744

[ref115] van der Does A. M. Hiemstra P. S. Mookherjee N. (2019). “Antimicrobial host Defence peptides: immunomodulatory functions and translational prospects” in Antimicrobial Peptides. ed. Matsuzaki K., vol. 1117 (Singapore: Springer Singapore), 149–171. 10.1007/978-981-13-3588-4_10 30980358

[ref116] van Groenendael R. Beunders R. Hofland J. Morshuis W. J. Kox M. van Eijk L. T.. (2019). The safety, tolerability, and effects on the systemic inflammatory response and renal function of the human chorionic gonadotropin hormone-derivative EA-230 following on-pump cardiac surgery (the EASI study): protocol for a randomized, double-blind, placebo-controlled phase 2 study. JMIR Res. Protocols 8:e11441. doi: 10.2196/11441 PMC6381408 30724734

[ref117] van Hoek M. (2014). Antimicrobial Peptides in Reptiles. Pharmaceuticals 7, 723–753. doi: 10.3390/ph7060723 24918867 PMC4078517

[ref118] von Köckritz-Blickwede M. Goldmann O. Thulin P. Heinemann K. Norrby-Teglund A. Rohde M.. (2008). Phagocytosis-independent antimicrobial activity of mast cells by means of extracellular trap formation. Blood 111, 3070–3080. doi: 10.1182/blood-2007-07-104018 18182576

[ref119] Waghu F. H. Barai R. S. Gurung P. Idicula-Thomas S. (2016). CAMP _R3_: A database on sequences, structures and signatures of antimicrobial peptides. Nucleic Acids Res. 44, D1094–D1097. doi: 10.1093/nar/gkv1051 26467475 PMC4702787

[ref120] Wang G. (2014). Human antimicrobial peptides and proteins. Pharmaceuticals 7, 545–594. doi: 10.3390/ph7050545 24828484 PMC4035769

[ref121] Wang F. Qiao L. Lv X. Trivett A. Yang R. Oppenheim J. J.. (2016). Alarmin human α defensin HNP1 activates plasmacytoid dendritic cells by triggering NF-κB and IRF1 signaling pathways. Cytokine 83, 53–60. doi: 10.1016/j.cyto.2016.03.015 27031443 PMC7822553

[ref122] Wei L. Gao J. Zhang S. Wu S. Xie Z. Ling G.. (2015). Identification and characterization of the first cathelicidin from sea snakes with potent antimicrobial and anti-inflammatory activity and special mechanism. J. Biol. Chem. 290, 16633–16652. doi: 10.1074/jbc.M115.642645 26013823 PMC4505416

[ref123] Wu Z. Hoover D. M. Yang D. Boulègue C. Santamaria F. Oppenheim J. J.. (2003). Engineering disulfide bridges to dissect antimicrobial and chemotactic activities of human β-defensin 3. Proc. Natl. Acad. Sci. 100, 8880–8885. doi: 10.1073/pnas.1533186100 12840147 PMC166407

[ref124] Xiao Y. Cai Y. Bommineni Y. R. Fernando S. C. Prakash O. Gilliland S. E.. (2006a). Identification and functional characterization of three chicken Cathelicidins with potent antimicrobial activity. J. Biol. Chem. 281, 2858–2867. doi: 10.1074/jbc.M507180200 16326712

[ref125] Xiao Y. Dai H. Bommineni Y. R. Soulages J. L. Gong Y.-X. Prakash O.. (2006b). Structure-activity relationships of fowlicidin-1, a cathelicidin antimicrobial peptide in chicken. FEBS J. 273, 2581–2593. doi: 10.1111/j.1742-4658.2006.05261.x 16817888

[ref126] Xu D. Lu W. (2020). Defensins: A double-edged sword in host immunity. Front. Immunol. 11:764. doi: 10.3389/fimmu.2020.00764 32457744 PMC7224315

[ref127] Xuan J. Feng W. Wang J. Wang R. Zhang B. Bo L.. (2023). Antimicrobial peptides for combating drug-resistant bacterial infections. Drug Resist. Updat. 68:100954. doi: 10.1016/j.drup.2023.100954 36905712

[ref128] Yang D. Chertov O. Bykovskaia S. N. Chen Q. Buffo M. J. Shogan J.. (1999). β-Defensins: linking innate and adaptive immunity through dendritic and T cell CCR6. Science 286, 525–528. doi: 10.1126/science.286.5439.525 10521347

[ref129] Yang Y. Jiang Y. Yin Q. Liang H. She R. (2010). Chicken intestine defensins activated murine peripheral blood mononuclear cells through the TLR4-NF-κB pathway. Vet. Immunol. Immunopathol. 133, 59–65. doi: 10.1016/j.vetimm.2009.07.008 19695713

[ref130] Yu J. Mookherjee N. Wee K. Bowdish D. M. E. Pistolic J. Li Y.. (2007). Host defense peptide LL-37, in synergy with inflammatory mediator IL-1β, augments immune responses by multiple pathways. J. Immunol. 179, 7684–7691. doi: 10.4049/jimmunol.179.11.7684 18025214

[ref131] Zasloff M. (1987). Magainins, a class of antimicrobial peptides from Xenopus skin: isolation, characterization of two active forms, and partial cDNA sequence of a precursor. Proc. Natl. Acad. Sci. 84, 5449–5453. doi: 10.1073/pnas.84.15.5449 3299384 PMC298875

[ref132] Zhao H. Gan T.-X. Liu X.-D. Jin Y. Lee W.-H. Shen J.-H.. (2008). Identification and characterization of novel reptile cathelicidins from elapid snakes. Peptides 29, 1685–1691. doi: 10.1016/j.peptides.2008.06.008 18620012

[ref133] Zheng Y. Niyonsaba F. Ushio H. Nagaoka I. Ikeda S. Okumura K.. (2007). Cathelicidin LL-37 induces the generation of reactive oxygen species and release of human α-defensins from neutrophils. Br. J. Dermatol. 157, 1124–1131. doi: 10.1111/j.1365-2133.2007.08196.x 17916212

