# Supplementary material for: Immunomodulatory peptides: new therapeutic horizons for emerging and re-emerging infectious diseases
Source: Front Microbiol. 2024 Dec 20;15:1505571. doi: 10.3389/fmicb.2024.1505571 (PMC11695410; doi:10.3389/fmicb.2024.1505571)
Supplement: Supplementary file 1 [file Table_1.DOCX]

**Table 1** List of host defence peptides from various sources along with their structural details and mechanism of action.

| **Name of the Peptide** | | | | **Sequence** | **Source** | **Number of amino acids/Mol.wt** | **Secondary Structure composition analysis using SOPMA** | **PDB** | **Mechanism of action** | **Reference** |
| --- | --- | --- | --- | --- | --- | --- | --- | --- | --- | --- |
| **Humans** | | | | | | | | | |  |
| β-defensins | | HBD1 | | DHYNCVSSGGQCLYSACPIFTKIQGTCYRGKACCK | Constitutively expressed by human epithelial cells. | 35/ 3806.4323 | Alpha Helix (45.71%)  Extended Strand(34.29%)  Beta turn(20.00%) | 1KJ5 | Chemoattractant for immature dendritic cells and memory T cells and mediates activity by a chemokine receptor CCR6. | (Valore et al., 1998)  (Hoover et al., 2001)  (Semple & Dorin, 2012) |
|  |  | HBD2 | | TCLKSGAICHPVFCPRRYKQIGTCGLPGTKCCKKP | Skin | 35/ 3795.6725 | Extended Strand: 2.86%  Beta turn: 5.71%  Random coil: 91.43% | 1FD3 | Pro-inflammatory; expressed in atopic dermatitis, GI infection at both mRNA and protein level. | (Schröder & Harder, 1999)  (Koeninger et al., 2020) |
| α-defensins | | HNP1 | | ACYCRIPACIAGERRYGTCIYQGRLWAFCC | Neutrophils | 30 / 3448.1296 | Alpha helix(3.33%); Extended strand (43.33%); Beta turn (20.00%) and Random coil (33.33%) 1 is 3.33% | 3GO0 | Pro-inflammatory and inhibits differentiation of monocytes; potent chemoattractant that HNP2 | (Brook et al., 2016)  (F. Wang et al., 2016)  (Bowdish et al., 2006) |
|  |  | HNP2 | | CYCRIPACIAGERRYGTCIYQGRLWAFCC | Neutrophils | 29 / 3377.0508 | Extended strand: 44.83%  Beta turn: 13.79%  Random coil: 41.83% | 1ZMH | Induces chemokine activity in monocytes | (Lehrer & Lu, 2012)  (G. Wang, 2014)  (Pachón-Ibáñez et al., 2017) |
|  |  | HNP4 | | CYCRIPACIAGERRYGTCIYQGRLWAFCC | Neutrophils | 29 / 3377.0508 | Extended strand: 44.83%  Beta turn: 13.79%  Random coil: 41.83% | 6DMM | found in neutrophils and have corticostatic activity. | (Xu & Lu, 2020)  (Bowdish et al., 2006) |
|  |  | HDP5 | | ATCYCRHGRCATRESLSGVCEISGRLYRLCCR | Paneth Cells of the small intestine | 32 / 3624.2628 | Alpha helix: 46.88%  Extended strand: 15.62%  Beta turn: 12.5%  Random coil: 25% | __ | Expressed at high concentration in jejunum and ileum, level increases during acute coeliac sprue and decreases in HIV | (Petkovic et al., 2021)  (Hancock et al., 2016) |
|  |  | HDP6 | | ATCYCRHGRCATRESLSGVCEISGRLYRLCCR | Paneth cell of small intestine | 32 / 3624.2628 | Alpha helix: 46.88%  Extended strand: 15.62%  Beta turn: 12.5%  Random coil: 25% | __ | Expressed at high concentration in jejunum and ileum and level increases during acute coeliac sprue and decreases in HIV. |  |
| Cathelicidin/LL-37 | | | | LLGDFFRKSKEKIGKEFKRIVQRIKDFLRNLVPRTES | Epithelial cells predominantly the cells of the skin and respiratory tract | 37 / 4493.3271 | Alpha helix (91.89%); Random Coil: 5.41%  Beta turn: 2.7% | 2FBS | Pro/Anti-inflammatory and allows chemotactic regulation of monocytes | (Sandra Tjabringa et al., 2005) (Nell et al., 2006)  (X. Chen et al., 2013) |
| **Plants** | | | | | | | | | | |
| Cyclotide Cliotide | | T28 | | GGSIPCGESCVFLPCFLPGCSCKSSVCYLN | *Clitoria ternatea* L. | 30 / 3071.65 | Extended strand: 40%  Random coil: 50%  Beta turn: 10% | __ | Invitro increase in anti-inflammatory interleukines IL-8, IL6, TNF-α in macrophages | (Gilding et al., 2016) |
|  |  | T32 | | GDLFKCGETCFGGTCYTPGCSCDYPICKNN |  | 30 / 3197.6440 | Extended strand: 6.67%  Beta turn: 13.33%  Random coil:80% | ___ |  | (Serra et al., 2016) |
|  |  | T33 | | GFNSCSEACVYLPCFSKGCSCFKRQCYKN |  | 29 / 3273.8345 | Extended strand: 24.14%  Beta turn: 17.24%  Random coil: 58.62% | __ |  | (Nguyen et al., 2011) |
| Zein hydrolysates | | Peptide 1 | | PFNQL | *Zea mays* L. | 5/ 617.7026 | Random coil: 100% | __ | Invitro analysis showed inhibition of interleukin IL—6 in human cell line U937 | (Liu et al., 2020) |
|  |  | Peptide 2 | | FLPFNQL |  | 7 / 878.0387 | Random coil: 100% | __ |  |  |
|  |  | Peptide 3 | | SQLALTNPT |  | 9 / 944.0527 | Random coil: 100% | __ |  |  |
|  |  | Peptide 4 | | GAPFNQ |  | 6 / 632.6740 | Random coil: 100% | __ |  |  |
|  |  | Peptide 5 | | FLPPVT |  | 6/ 672.8223 | Random coil: 100% | __ |  |  |
| LR13 | | | | LLPPFHQASSLL | *Oryza sativa* L. | 12/ 1322.5704 | Random coil: 100% | __ | Invitro and invivo anti-inflammatory response. Invitro downregulated IL-1β expression in macrophages, invivo upregulated IL-4 and IL-10 in CD4+ and CD25+ cells | (Shapira et al., 2010) |
| α-Gliadin (pepsin-trypsin digested fragment) | | | | PPYCTIVPFGIFGTNYR | Glutein containing grains | 17 / 1945.2725 | Extended strand: 29.41%  Random coil: 70.59% | __ | Bind to chemokine receptor CXCR3 and induced the release of IL-8 in coeliac patients | (Lammers et al., 2011) |
| **Amphibians- Frogs** | | | | | | | | | | |
| Frenatin-2D | | | | DLLGTLGNLPLPFI | *Discoglossus sardus* (Tyrrhenian painted frog) | 14 / 1482.7838 | Random coil: 100% | __ | Mouse peritoneal macrophages were stimulated by the peptide to release proinflammatory cytokines TNF- and IL-1.Also, both LPS-stimulated and unstimulated cells were stimulated for the production of IL-12. | (Conlon et al., 2013) |
| Plasticin-L1 | | | | GLVNGLLSSVLGGGQGGGGLLGGIL | *Leptodactylus laticeps* (South-American Santa Fe frog) | 25 | Solvent-dependent secondary structure. In water it exhibits random coil, in methanol it exhibits beta sheet structure and mixture of 50% trifluoroethanol and water, it exhibits an alpha-helical conformation. | __ | Peritoneal macrophages from C57BL/6 and BALB/c mice produced increased proinflammatory interleukins IL-1β, IL-12, IL-23, and TNF-α. | (Scorciapino et al., 2013) |
| Pseudhymenochirin | 1Pb | | | IKIPSFFRNILKKVGKEAVSLIAGALKQS | *Pseudhymenochirus merlini* | 29 / 3156.8505 | Alpha helix: 75.86%  Beta turn: 13.79%  Random coil: 10.34% | __ | Upregulation of proinflammatory IL-23 production and downregulation of anti-inflammatory IL-6 and IL-10 production by LPS-stimulated macrophages from mice | (Mechkarska et al., 2014) |
|  | 2Pa | | | GIFPIFAKLLGKVIKVASSLISKGRTE |  | 27 / 2873.5199 | Alpha helix: 70.37%  Beta turn: 11.11%  Random coil: 18.52% | __ |  |  |
| Magainin-AM1 | | | | GIKEFAHSLGKFGKAFVGGILNQ | *Xenopus amieti* (African volcano frog) | 23/ 2418.8253 | Alpha helix: 78.26%  Extended strand: 8.7%  Beta turn: 13.04% | __ | Stimulated enhanced production of pro-inflammatory interleukin IL-8 by oral fibroblasts | (McLean et al., 2014) |
| Esculentin 2CHa | | | | GFSSIFRGVAKFASKGLGKDLAKLGVDLVACKISKQC | *Lithobates chiricahuensis* (Chiricahua leopard frog) | 37/ 3843.6182 | Alpha helix: 75.68%  Extended strand: 8.11%  Beta turn: 8.11%  Random coil: 8.11% | __ | Enhanced the release and production of anti-inflammatory IL-10 by mouse lymphoid cells and also increase production of TNFα by peritoneal macrophages | (Attoub et al., 2013) |
| Brevinin-2GU | | | | GVIIDTLKGAAKTVAAELLRKAHCKLTNSC | Hylarana guentheri | 30 / 3125.7520 | Alpha helix: 76.67%  Extended strand: 10%  Beta turn: 6.67%  Random coil: 6.67% | __ | Downregulated the production of TNFα from Con A stimulated peripheral mononuclear cells and IFN-γ production in unstimulated cells | (Popovic et al., 2012) |
| Temporin | | | | HFLGKLVNLAKKIL | R. draytonii | 14 / 1594.0203 | Random coil: 100% | __ | Upregulation of IL-10, IL-4, TGF-β from treated and untreated cells | (Mangoni, 2006) |
| Tigirin | | | IR | RVCSAIPLPICH |  | 12 / 1308.6351 | Random coil: 100% | __ | Stimulated human peripheral blood mononuclear cells, as well as mouse peritoneal macrophages and splenocytes for increased the production of the anti-inflammatory cytokine IL-10 in both LPS-stimulated and unstimulated cells. | (Ojo et al., 2013) |
|  | | | IV | RICYAMWIPYPC | *Lithobates vaillanti* | 12/ 1515.8875 | Random coil: 100% | __ |  | (Conlon et al., 2009) |
|  | | | IM | WCPPMIPLCSRF | *Xenopus muelleri* | 12 / 1449.8282 | Random coil: 100% | __ |  | (Ali et al., 2001) |
| **Marine Organisms** | | | | | | | | | | |
| Mytilus protein hydrolysate peptide | | | | GVSLLQQFFL | *Mytilus coruscus* (Shell fish) | 10 / 1151.3713 | Random coil: 100% | __ | Inhibited LPS-induced NO production in RAW264.7 macrophages | (E.-K. Kim et al., 2013) |
| Tilapapiscidin peptides | | | TP3 | FIHHIIGGLFSVGKHIHSLIHGH | *Oreochromis niloticus* (Nile tilapa; cichild fish) | 23 / 2557.0033 | Alpha helix: 47.83%  Extended strand: 21.74%  Beta turn: 13.04%  Random coil: 17.39% | __ | Significantly increased the expression of several immune-related genes in muscle (IL-1β, IL-6, IL-8 TGF-β, and IκB) and decreased the expression of Toll-like receptor 5 (TLR5) to combat aquaculture bacterial pathogens | (Lin et al., 2016) |
|  |  |  | TP4 | FIHHIIGGLFSAGKAIHRLIRRRRR |  | 25 / 2981.6001 | Alpha helix: 72%  Extended strand: 12%  Beta turn: 12%  Random coil: 4% | __ |  |  |
| Phosvitin-derived peptide Pt5 | | | | SRMSKTATIIEPFRKFHKDRYLAHHSATKDTSSGSAAASFEQMQKQNRFLGNDIP | Zebra fish | 55 / 6240.0122 | Alpha helix: 50.91  Extended strand: 3.64%  Beta turn: 7.27%  Random coil: 38.18% | __ | inhibits the expression of proinflammatory cytokine genes (IL-1β, IL-6, TNF-α, and IFN-γ) in the spleen and head kidneys of A. hydrophila-infected zebrafish, but increased the expression of anti-inflammatory cytokine genes (IL-10 and IL-14) | (Ding et al., 2012) |
| Clavanin | A | | | VFQFLGKIIHHVGNFVHGFSHVF | *Styela clava* (TUNICATA) | 23 / 2667.1150 | Alpha helix: 86.96%  Random coil: 13.04% | 6C41 | increased the level of IL-10, an anti-inflammatory cytokine, and decreased the levels of IL-12 and TNF-α, two pro-inflammatory cytokines that boost inflammation and may lead to excessive damage | (Lee et al., 1997) |
|  | MO | | | FLPIIVFQFLGKIIHHVGNFVHGFSHVF |  | 28 / 3250.8868 | Alpha helix: 67.86%  Extended strand: 10.71%  Random coil: 21.43% | __ |  | (Silva et al., 2016) |
| **Birds** | | | | | | | | | | |
| β-defensins | AvBD2 | | | LFCKGGSCHFGGCPSHLIKVGSCFGFRSCCKWPWNA | Chicken | 36/ 3921.61 | ___ | __ | Chemokine, CD4+ and CD8+ -T-cells and B-lymphocytes were chemotaxic towards peptide invitro. Downregulated C-type lecithin receptor in splenocytes | (Soman et al., 2009) |
|  | AvBD13 | | | MRILQLLFAIVVILLLQDAPARGFSDSQLCRNNHGHCRRLCFHMESWAGSCMNGRLRCCRFSTKQPFSNP  KHSVLHTAEQDPSPSLGGT | Chicken | 65/ 9996.52 | ___ | __ | Activated NF-κB cells, stimulated IL-12 and IFN-α production, and elevated CD80 and monocyte proliferation in murine PBMC cells, | (Y. Yang et al., 2010) |
| Cathelicidins | Cath-1 (Fowlicidin-1) | | | RVKRVWPLVIRTVIAGYNLYRAIKKK | Chicken | 26 / 3141.8909 | Alpha helix: 76.92%  Beta turn: 3.85%  Random coil: 19.23% | 2AMN | Inhibited LPS-induced macrophage activation thereby inhibiting MCP-1,TNF α, IL-1α, and NO production in RAW264.7 mouse macrophages | (Xiao, Dai, et al., 2006)  (Bommineni et al., 2007)  (Xiao, Dai, et al., 2006) |
|  | Cath-3 (Fowlicidin—3) | | | KRFWPLVPVAINTVAAGIN LYKAIRRK | Chicken | 27/ 3095.7753 | Alpha helix: 55.56%  Extended strand: 11.11%  Beta turn: 3.7%  Random coil: 29.63% | 2HFR |  |  |
|  | d-CATH | | | KRFWQLVPLAIKIYRAWKRR | Shaoxing ducks, Anas platyrhynchos | 20/ 2629.2431 | Alpha helix: 80.00%  Beta turn:10%  Random coil : 10% | ___ | Anti-inflammatory effect: binds to LPS of bacterial cells | (Feng et al., 2020) |
| **Reptiles** | | | | | | | | | | |
| Cathelicidins | Cathelicidin-BF | | | KRFKKFFRKLKKSVKKRAKEFFKKPRVIGVSIPF | *Bungarus fascia (Branded krait snake)* | 34/ 4198.19 | Alpha helical conformation | __ | Invitro inhibited STAT and NF-κB pathway, thereby downregulating the production of pro-inflammatory cyotkines and enhanced the production of anti-inflammatory cytokines IL-4, IL-10 in *E. coli* K88-induced macrophages | (S. Chen et al., 2018) |
|  | Cm-CATH2 | | | RRSRFGRFFKKVRKQLGRVLRHSRITVGGRMRF | *Chelonia mydas* (green sea turtle) | 33 / 4089.9366 | Alpha helix: 60.61%  Beta turn: 15.15%  Random coil: 18.18% | __ | Blocked TLR4/MD2 complex and the downstream signalling pathway activation, which increased the trafficking of neutrophils, macrophages, and monocytes to the infection site and reduced the generation of inflammatory cytokines caused by LPS. | (Qiao et al., 2019) |
|  | Hc-CATH | | | KFFKRLLKSVRRAVKKFRKKPRLIGLSTLL | *Hydrophis cyanocinctus (sea snake)* | 30 / 3628.5931 | Alpha helix: 60%  Extended strand: 16.67%  Random coil: 23.33% | __ | Exhibited anti-inflammatory activity by downregulating the LPS-induced NO and pro-inflammatory cytokines such as TNF-α, IL-1β, and IL-6 production | (Wei et al., 2015) |
