# Supplementary material for: Immunomodulatory peptides: new therapeutic horizons for emerging and re-emerging infectious diseases
Source: Front Microbiol. 2024 Dec 20;15:1505571. doi: 10.3389/fmicb.2024.1505571 (PMC11695410; doi:10.3389/fmicb.2024.1505571)
Supplement: Supplementary file 2 [file Table_2.DOCX]

**Table 2** List of immunomodulatory peptides under clinical trails

| **Name of the peptide** | **Derived from** | **Developed by** | **Immunomodulatory activity** | **Clinical trial phase** | **Reference** |
| --- | --- | --- | --- | --- | --- |
| EA230 | Beta-chain of human gonadotropin | Exponential biotherapeutics | Upregulation of pro-inflammatory cytokines and neutrophil efflux | Phase II | (van Groenendael et al., 2019) |
| CZEN-002 | α-melanocyte-stimulating hormone | Zengen | Downregulation of TNF-α production | Phase II | (Duncan & O’Neil, 2013) (Fjell et al., 2012) |
| Delmitide (RDP 58) | HLA class I. | Genzyme | inhibition pro-inflammatory cytokines synthesis | Phase II | (Travis et al., 2005) |
| Ghrelin | Host defense peptide (endogenous) | Royal Papworth Hospital (Cambridge, UK) | treatment of airway inflammation, chronic respiratory and lung infection | Phase II | (Min et al., 2012) (Mookherjee et al., 2012) |
| Dusquetide (SGX942) | - | Soligenix | Modulation of innate immunity | Phase III | (Kudrimoti et al., 2016) |
